# Machine Learning–Based Prediction for High Health Care Utilizers by Using a Multi-Institutional Diabetes Registry: Model Training and Evaluation

**DOI:** 10.2196/58463

**Published:** 2024-10-17

**Authors:** Joshua Kuan Tan, Le Quan, Nur Nasyitah Mohamed Salim, Jen Hong Tan, Su-Yen Goh, Julian Thumboo, Yong Mong Bee

**Affiliations:** 1 Health Services Research Unit Singapore General Hospital Singapore Singapore; 2 Data Science and Artificial Intelligence Laboratory Singapore General Hospital Singapore Singapore; 3 Department of Endocrinology Singapore General Hospital Singapore Singapore

**Keywords:** diabetes mellitus, type 2 diabetes, health care utilization, population health management, population health, machine learning, artificial intelligence, predictive model, predictive system, practical model

## Abstract

**Background:**

The cost of health care in many countries is increasing rapidly. There is a growing interest in using machine learning for predicting high health care utilizers for population health initiatives. Previous studies have focused on individuals who contribute to the highest financial burden. However, this group is small and represents a limited opportunity for long-term cost reduction.

**Objective:**

We developed a collection of models that predict future health care utilization at various thresholds.

**Methods:**

We utilized data from a multi-institutional diabetes database from the year 2019 to develop binary classification models. These models predict health care utilization in the subsequent year across 6 different outcomes: patients having a length of stay of ≥7, ≥14, and ≥30 days and emergency department attendance of ≥3, ≥5, and ≥10 visits. To address class imbalance, random and synthetic minority oversampling techniques were employed. The models were then applied to unseen data from 2020 and 2021 to predict health care utilization in the following year. A portfolio of performance metrics, with priority on area under the receiver operating characteristic curve, sensitivity, and positive predictive value, was used for comparison. Explainability analyses were conducted on the best performing models.

**Results:**

When trained with random oversampling, 4 models, that is, logistic regression, multivariate adaptive regression splines, boosted trees, and multilayer perceptron consistently achieved high area under the receiver operating characteristic curve (>0.80) and sensitivity (>0.60) across training-validation and test data sets. Correcting for class imbalance proved critical for model performance. Important predictors for all outcomes included age, number of emergency department visits in the present year, chronic kidney disease stage, inpatient bed days in the present year, and mean hemoglobin A_1c_ levels. Explainability analyses using partial dependence plots demonstrated that for the best performing models, the learned patterns were consistent with real-world knowledge, thereby supporting the validity of the models.

**Conclusions:**

We successfully developed machine learning models capable of predicting high service level utilization with strong performance and valid explainability. These models can be integrated into wider diabetes-related population health initiatives.

## Introduction

In recent years, high-income countries worldwide have seen a consistent rise in health care expenditure. Singapore, mirroring this trend, has experienced a steady increase in health care spending relative to its gross domestic product [1]. To address this, Singapore is undergoing a transformative health system initiative known as Healthier SG [2], which is an initiative to pivot the health system toward preventive care and population health management.

Parallel to these efforts, there is a burgeoning interest in leveraging machine learning for individual-level health utilization predictions. Identifying prospective high utilizers of health care services could unlock opportunities for targeted interventions. These interventions are poised not only to enhance individual health outcomes but also to reduce long-term health care utilization and system costs. Existing research suggests that a disproportionate amount of health care spending is concentrated among a small group of costly patients known as the high-need, high-cost (HNHC) patients—often defined as those who account for the top 5% of the annual health care costs [3,4]. These patients were believed to present an opportunity for cost reduction [5].

However, the potential for cost savings in caring for HNHC patients is often less than anticipated [6]. This is due to the diverse nature of these patients who can be subdivided into 3 categories: persistent and refractory HNHC patients, individuals who experience a 1-time catastrophic health event, and patients with multiple chronic conditions but amenable to disease management programs [6,7]. Notably, the latter group presents the most viable opportunity for impactful intervention. Persistent and refractory HNHC patients are those with severe and chronic diseases who require ongoing and expensive care. For these patients, disease management programs often do not result in significant reduction in health utilization and financial savings. For patients with 1-time catastrophic health events such as accidents, these events are difficult to predict and therefore not amenable to any intervention [6,7]. Therefore, targeting the small cohort with multiple chronic conditions but amenable to disease management programs represents a limited opportunity to reduce health care costs [6].

Given these complexities, there is a need to refine the approach to predicting and managing high health care utilization. One strategy could be to expand the predictive scope beyond HNHC patients or explore other indicators. Relatedly, the total length of stay (LOS) and frequency of emergency department (ED) visits per calendar year may provide a better indication of service-related health care utilization and the intensity of inpatient resource use [8].

This study aims to develop prediction models to forecast annual inpatient bed days and ED utilization across varying thresholds; presently, such models are not available in our hospital system. We utilized the Singapore Health Services (SingHealth) Diabetes Registry (SDR), a comprehensive clinical database of patients with diabetes within our hospital system to develop predictive models. Our objective is to create clinically relevant and actionable models that can be integrated into wider diabetes-related population health initiatives [9].

## Methods

### Study Setting

We used data from the multi-institutional SDR, previously described in detail [10]. SingHealth is the largest of the 3 public health care clusters in Singapore and manages 4 acute hospitals, 5 national specialty centers, 3 community hospitals, and a network of 10 primary care polyclinics. SDR was initiated in 2015 and populated retrospectively and prospectively from across SingHealth’s electronic medical records and clinical databases to cover the period of 2013 to 2022.

### Outcome Variables

As SDR primarily consists of clinical data from electronic medical records and lacks financial information, we focused on service-related health care utilization metrics. To this end, we developed models to predict utilization across 6 different thresholds (per calendar year), specifically for total LOS at ≥7, ≥14, and ≥30 days and for ED attendance ≥3, ≥5, and ≥10 visits; thus, 6 sets of (binary classification) models were constructed. Currently, there are no standard definitions for long inpatient LOS or high ED attendance.

For total LOS, we set arbitrary thresholds corresponding to 1 week, 2 weeks, and 1 month. These thresholds were chosen to reflect varying degrees of health care utilization in ours and possibly other health care systems, corresponding to different levels of patient care needs and resource allocation. Inpatient stays between 1 and 2 weeks represent short-term stays, potentially indicative of acute or less severe conditions. In contrast, stays longer than 2 weeks and those extending beyond 1 month represent increasingly prolonged stays, often associated with more severe or complex health issues, especially in the latter. These distinctions are critical for understanding and managing different patient care strategies. They also represent varying levels of health care management and resource planning, as we intend to develop disease management programs around these thresholds in the future. Regarding ED attendance, a recent systematic review indicated that ≥3 was the most common definition for high ED attendance but noted that definitions could extend to 30 or more visits [11]. Accordingly, we defined high ED attendance by using the 3 aforementioned thresholds, with ≥3 visits as the minimum criterion. This approach may aid in planning interventions to prevent escalation to higher levels of utilization.

### Explanatory Variables

The SDR data set facilitated an examination of the effects of sociodemographic indicators, health indicators, and diabetes-related complications. Our methodology for ascertaining diabetes-related complications has been published previously [12] and detailed in Table S1 of Multimedia Appendix 1. The models incorporated 24 variables detailed in Table S2 of Multimedia Appendix 1. These variables are readily derived from electronic medical records during admissions, ED visits, inpatient and outpatient clinical consultations, and are based on local clinical guidelines [13]. These variables offer a comprehensive view of the patients from demographic, social, clinical, and utilization perspectives.

### Inclusion and Exclusion Criteria

This study utilizes data from SDR spanning 2019 to 2022, as this was the period when comprehensive health care utilization data were available. We included patients aged 18 years and older diagnosed with type 2 diabetes mellitus. Patients with missing variables were excluded from this study, as we did not perform data imputation, and most machine learning algorithms do not support missing values.

### Handling Unbalanced Data

Our data set demonstrated significant class imbalance in inpatient and ED utilization, which can bias models toward the majority class, hinder the identification of the high utilizers (the minority class) [14], and result in subpar model performance. In this study, we utilized oversampling, a data-level method to address the class imbalance. Specifically, we used the synthetic minority oversampling technique-nominal continuous (SMOTE-NC) [15] from the *themis* package [16]. SMOTE-NC, a variant of the SMOTE family of algorithms, generates new examples of the minority class by interpolating between several minority class instances that lie relatively close to each other [17]. SMOTE-NC is effective with mixed numerical and categorical data. We applied SMOTE-NC with k=5 and k=3 settings, where k denotes the number of nearest neighbors used to generate new examples of the minority class. Additionally, we used the upSample algorithm from the *caret* package [18] for random oversampling and compared it with no oversampling. All oversampling techniques achieved equal representations of both classes in our training data set (ie, equal number of patients with and without the outcome in the training data set).

### Performance Indicators

We assessed model performance by using area under the receiver operating characteristic curve (AUC), sensitivity (recall), and positive predictive value (PPV). Sensitivity (recall) allowed us to identify whether the models were able to correctly identify patients with the outcomes of interest. PPV provided us with an understanding of the quality of the positive prediction made by the model. Additionally, we have reported the area under the precision-recall curve, sensitivity, specificity, and *F*_1_-score in Multimedia Appendix 1. The area under the precision-recall curve is preferred over AUC for rare outcomes, as it more accurately reflects model performance [19]. We also evaluated the confusion matrix during model development.

### Machine Learning Models

We built 7 predictive models using R software (version 4.3.1; R Foundation for Statistical Computing) and the *tidymodels* package [20]: logistic regression, random forest, boosted trees, multilayer perceptron (MLP), k-nearest neighbor, multivariate adaptive regression splines (MARS), and Bayesian additive regression trees. SDR data from 2019 were randomly split into training (75%) and validation (25%) data sets, with no overlap between the data sets. Since the training data set was large (n=75,375), we did not perform cross-validation during model training. No hyperparameter tuning was performed, as the intent of the study was to build baseline models to understand the problem and data set while prioritizing model simplicity and interpretability. The trained models were then tested on unseen data from 2020 and 2021 (ie, the model utilized 2020 data to predict 2021 outcomes and 2021 data to predict 2022 outcomes). Although the data sets originate from the same registry, they reflect distinct utilization patterns across different years, ensuring temporal independence between them.

### Explainability

For top-performing models, model interpretation was determined using model-specific variable importance scores with the *vip* package [21] and permutation feature importance plots using the *DALEX* package [22,23]. Additionally, for the top variables identified through these methods, partial dependence plots (PDPs) were generated using the *DALEX* package and the unseen validation data set to visualize the relationship between key predictor variables and the probability of the outcome occurring.

### Ethics Approval

Ethics approval was obtained from the SingHealth Centralized Institutional Review Board prior to initiating this study (reference: 2022/2133). As all participant data were deidentified, a waiver for participant consent was also obtained.

### Reporting Checklist

We followed the consolidated reporting guidelines for prognostic and diagnostic machine learning modeling studies [24] (Table S3 in Multimedia Appendix 1).

## Results

### Characteristics of the Data Sets

After removing patients with missing data from the registry in 2019, the training data set contained 100,500 (74.6%) individuals of the 134,670 patients in SDR in 2019. The test sets in 2020 and 2021 comprised 77.3% (108,886/140,859) and 80.7% (111,004/137,584) of the total SDR cohorts for the respective years. The characteristics of the patients included in the training-validation and 2 test data sets are described in detail in Table 1.

**Table 1:** Demographics, comorbidities, and utilization characteristics of the training and test data sets.

Data set description				Training and validation^a^ 2019-2020 (n=134,670)			Test 2020-2021 (n=140,859)		Test 2021-2022 (n=137,584)
Data set size, n (% of total registry)				100,500 (74.6)			108,886 (77.3)		111,004 (80.7)
Female gender, n (%)				48,887 (48.6)			52,210 (48)		53,148 (47.9)
**Age on January 1 at the start of the year (years)**									
	Mean (SD)		66.4 (11.8)			66.7 (11.9)		66.5 (12.2)	
	Median		67			67		67	
**Ethnicity, n (%) **									
	Chinese		71,132 (70.8)			76,479 (70.2)		76,627 (69)	
	Malay		14,903 (14.8)			16,277 (15)		17,144 (15.4)	
	Indian		10,119 (10.1)			11,267 (10.4)		11,788 (10.6)	
	Other		4346 (4.3)			4863 (4.5)		5445 (4.9)	
**Housing type, n (%) **									
	1- and 2-room public housing		7502 (7.5)			8214 (7.5)		10,086 (9.1)	
	3-room public housing		24,976 (24.9)			26,741 (24.6)		24,779 (22.3)	
	4-room public housing		32,089 (31.9)			34,933 (32.1)		36,540 (32.9)	
	5-room public housing and executive flats		25,769 (25.6)			27,942 (25.7)		29,220 (26.3)	
	Private condominium		6268 (6.2)			6843 (6.3)		6607 (6)	
	Private landed housing		3896 (3.9)			4213 (3.9)		3772 (3.4)	
	Lives in a rental block		6641 (6.6)			7290 (6.7)		7294 (6.6)	
**Comorbidities, n (%) **									
	Hypertension				87,931 (87.5)	97,149 (89.2)		99,597 (89.7)	
	Hyperlipidemia				95,679 (95.2)	105,108 (96.5)		107,638 (97)	
**Diabetes mellitus medications, n (%)**									
	None				18,125 (18)	20,712 (19)		18,426 (16.6)	
	Oral medications only				57,413 (57.1)	64,571 (59.3)		61,516 (55.4)	
	Insulin only				2809 (2.8)	2264 (2.1)		3216 (2.9)	
	Oral and insulin				22,153 (22)	21,339 (19.6)		27,846 (25.1)	
**Diabetes-related complications, n (%) **									
	Ischemic heart disease				25,097 (25)	27,663 (25.4)		30,656 (27.6)	
	Ischemic stroke				9401 (9.4)	10,563 (9.7)		11,305 (10.2)	
	Hemorrhagic stroke				1449 (1.4)	1801 (1.7)		1998 (1.8)	
	Peripheral arterial disease				3910 (3.9)	4577 (4.2)		5198 (4.7)	
	Major lower-extremity amputation				138 (0.1)	173 (0.2)		182 (0.2)	
	Minor lower-extremity amputation				339 (0.3)	340 (0.3)		426 (0.4)	
	Diabetic foot and peripheral angiopathy				2718 (2.7)	3180 (2.9)		3524 (3.2)	
	Diabetic eye complications				13,067 (13)	13,116 (12.1)		14,479 (13)	
	Nephropathy				49,139 (48.9)	53,737 (49.4)		54,359 (49)	
	**Chronic kidney disease stage, n (%)**								
		1 (eGFR^b^ ≥90)			35,176 (35)	36,603 (33.6)		37,188 (33.5)	
		2 (eGFR 60-89)			41,705 (41.5)	45,216 (41.5)		45,755 (41.2)	
		3A (eGFR 45-59)			11,563 (11.5)	12,667 (11.6)		12,802 (11.5)	
		3B (eGFR 30-44)			6760 (6.7)	7696 (7.1)		7835 (7.1)	
		4 (eGFR 15-29)			3215 (3.2)	3805 (3.5)		4016 (3.6)	
		5 (eGFR<15)			2081 (2.1)	2899 (2.7)		3408 (3.1)	
	Dialysis				1400 (1.4)	1903 (1.8)		2269 (2)	
**Utilization characteristics**									
	**Inpatient utilization (present year)**								
		Mean (SD)			3.09 (11.3)	3.41 (11.7)		3.96 (13.6)	
		Median			0	0		0	
	**Inpatient bed days (present year), n (%)**								
		0			77,170 (76.8)	81,559 (74.9)		80,770 (72.8)	
		1-2			6034 (6)	6752 (6.2)		7168 (6.5)	
		3-6			6693 (6.7)	7701 (7.1)		8500 (7.7)	
		7-13			4464 (4.4)	5432 (5)		5982 (5.4)	
		14-29			3592 (3.6)	4315 (4)		4855 (4.4)	
		≥30			2547 (2.5)	3127 (2.9)		3729 (3.4)	
	**Inpatient bed days (subsequent year)**								
		Mean (SD)			2.39 (10.3)	2.79 (12.2)		3.22 (14)	
		Median			0	0		0	
	**Inpatient bed days category (subsequent year), n (%)**								
		0			83,759 (83.3)	90,022 (82.7)		89,577 (80.7)	
		1-2			4078 (4.1)	4214 (3.9)		4561 (4.1)	
		3-6			4477 (4.5)	5015 (4.6)		5619 (5.1)	
		7-13			3353 (3.3)	3729 (3.4)		4292 (3.9)	
		14-29			2740 (2.7)	3222 (3)		3722 (3.4)	
		≥30			2093 (2.1)	2684 (2.5)		3233 (2.9)	
**Emergency department utilization (present year)**									
	Mean (SD)		0.53 (1.4)			0.54 (1.4)		0.57 (1.6)	
	Median		0			0		0	
	**Emergency department visit category (present year), n (%)**								
		0 visits			71,584 (71.2)	76,261 (70)		75,376 (67.9)	
		1-2 visits			23,487 (23.4)	27,143 (24.9)		29,671 (26.7)	
		3-4 visits			3883 (3.9)	3938 (3.6)		4343 (3.9)	
		5-9 visits			1348 (1.3)	1358 (1.3)		1403 (1.3)	
		≥10 visits			198 (0.2)	186 (0.2)		211 (0.2)	
**Emergency department utilization (subsequent year)**									
	Mean (SD)		0.40 (1.3)			0.40 (1.4)		0.48 (1.4)	
	Median		0			0		0	
	**Emergency department visit category (subsequent year), n (%) **								
		0 visits			78,849 (78.5)	85,162 (78.2)		82,269 (74.1)	
		1-2 visits			17,794 (17.7)	19,434 (17.9)		23,273 (21)	
		3-4 visits			2716 (2.7)	3060 (2.8)		3817 (3.4)	
		5-9 visits			996 (1)	1064 (1)		1455 (1.3)	
		≥10 visits			145 (0.1)	166 (0.2)		190 (0.2)	

^a^The data set was randomly partitioned into training and validation data sets in a 75% to 25% ratio (respectively), with no overlap between the 2 data sets. n=total registry size.

^b^eGFR: estimated glomerular filtration rate in mL/min/1.73 m^2^.

Across the data sets, 47.9%-48.6% of the patients were females. The mean age was between 66.4 and 66.7 years, and the median was consistently 67 years. The proportions by ethnicities were consistent across the 3 data sets with approximately 70% Chinese, 14% Malay, 10% Indian, and 4% other races. The ethnic distributions observed closely resembled the Singaporean population [25]. Across the data sets, most individuals lived in public housing, with the largest proportion being 4-room public housing (approximately 32%). Owing to the public housing infrastructure in Singapore, approximately 6.6% of the patients live in an apartment block with rental housing. Across the data sets, the proportion of patients with hypertension was 87.5%-89.7%, whereas the proportion of patients with hyperlipidemia was 95.2%-97%. The most common diabetes-related complication was nephropathy (prevalence of 48.9%-49.4% across the data sets) followed by ischemic heart disease (prevalence of 25%-27.6%) and then diabetic eye complications (prevalence of 12.1%-13%). Relatedly, 65%-66.5% of the patients in the data sets had stage 2 chronic kidney disease (CKD) and above. When contrasted with the prevalence of nephropathy (our definition of nephropathy was estimated glomerular filtration rate <60 mL/min/1.73 m^2^ or urine albumin creatinine ratio ≥30 mg/g or urine protein/creatinine ratio ≥0.20 g/g), it suggests that a significant proportion of patients had stage 1 CKD and proteinuria.

The mean present year inpatient utilization across the data sets was 3.08%-3.96%. Compared to the present year, the subsequent year’s inpatient utilization was less. The mean present year ED utilization was 0.53-0.57 visits per patient. Compared to the present year, the subsequent year’s ED utilization was less. The median utilization for present and next year’s inpatient and ED utilization was zero across all data sets, indicating that the utilization characteristics were extremely skewed.

### Effects of Sampling Technique on Model Performance

**Figure 1:**
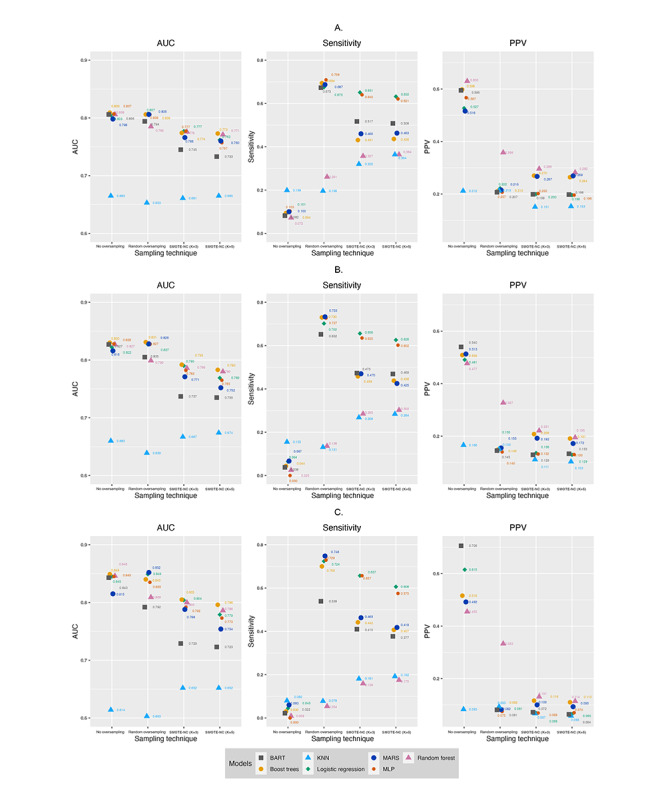
Comparing between different oversampling techniques to predict inpatient bed days. A. Predicting ≥7 inpatient bed days in subsequent year. B. Predicting ≥14 inpatient bed days in subsequent year. C. Predicting ≥30 inpatient bed days in subsequent year. AUC: area under the receiver operating characteristic curve; BART: Bayesian additive regression trees; KNN: k-nearest neighbor; MARS: multivariate adaptive regression splines; MLP: multilayer perceptron; PPV: positive predictive value; SMOTE-NC: synthetic minority oversampling technique-nominal continuous. A higher-resolution image of this figure is available in Multimedia Appendix 2.

**Figure 2:**
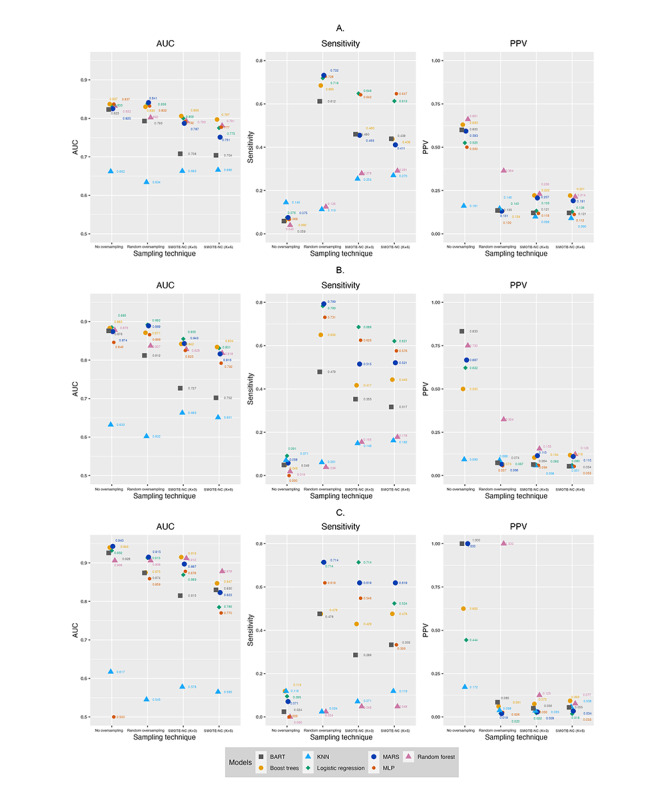
Comparing between different oversampling techniques to predict emergency department visits. A. Predicting ≥3 emergency department visits in subsequent year. B. Predicting ≥5 emergency department visits in subsequent year. C. Predicting ≥10 emergency department visits in subsequent year. AUC: area under the receiver operating characteristic curve; BART: Bayesian additive regression trees; KNN: k-nearest neighbor; MARS: multivariate adaptive regression splines; MLP: multilayer perceptron; PPV: positive predictive value; SMOTE-NC: synthetic minority oversampling technique-nominal continuous. A higher-resolution image of this figure is available in Multimedia Appendix 2.

The key model performance indices for the models using different oversampling techniques and no oversampling are presented in Figures 1-2 (Figures 1-2 in Multimedia Appendix 2) and Table S4 in Multimedia Appendix 1. For all the outcomes studied, models trained with random oversampling had similar AUC values to models trained with no oversampling, models trained with SMOTE-NC (k=3) had lower AUC values, and models trained with SMOTE-NC (k=5) had the lowest AUC. With regard to sensitivity, models trained with no oversampling had markedly lower sensitivity but higher PPVs. This indicates that models trained with no oversampling could not correctly identify patients with the outcomes of interest. This is further confirmed in our analysis of the confusion matrixes of these models trained. We observed that these models assigned almost all the patients as not cases (ie, did not have the outcomes the next year) and therefore were not useful. Models trained with no oversampling and SMOTE-NC (k=5) were not included in further analyses.

### Model Performance on Test Data Sets

**Figure 3:**
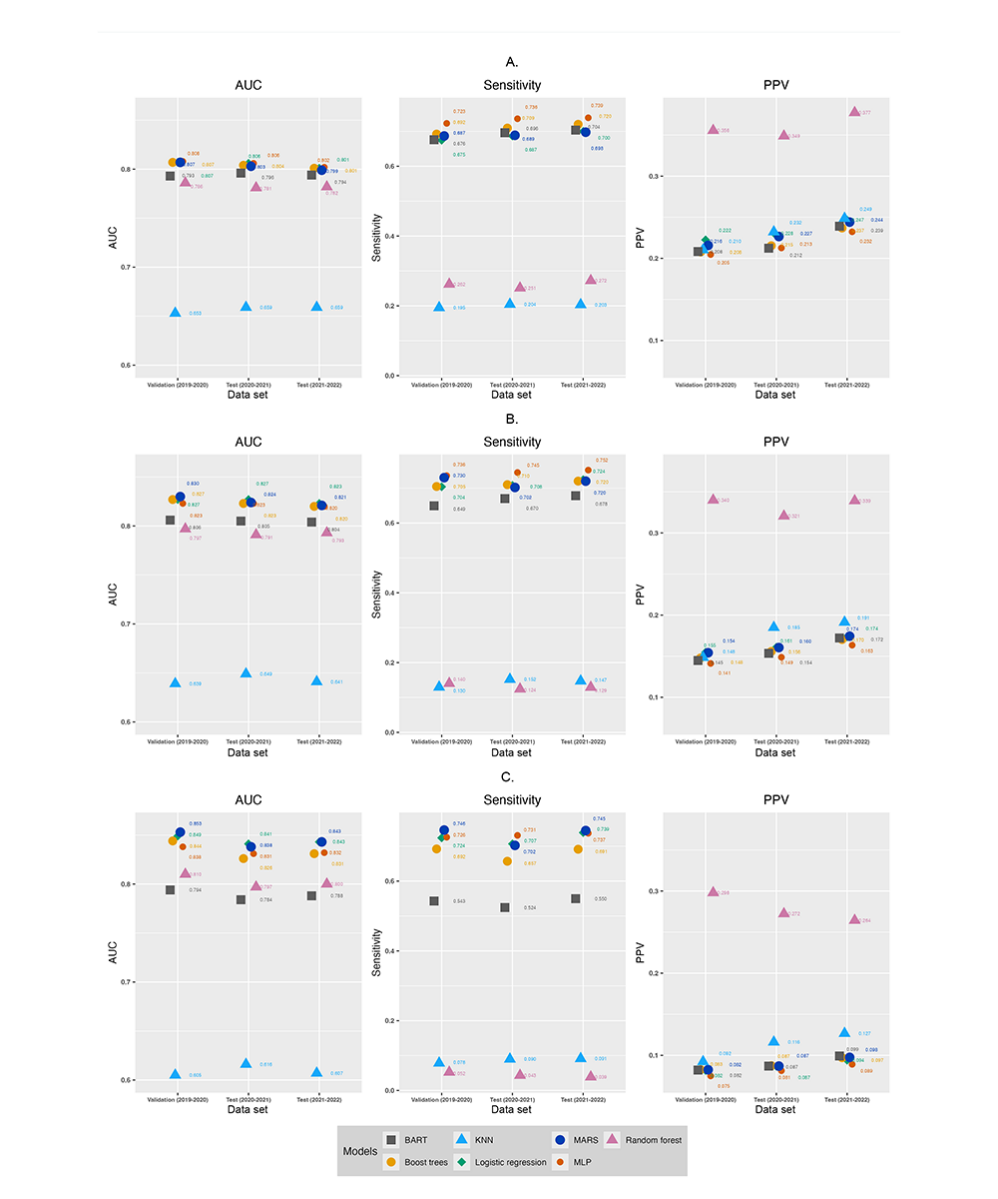
Performance of models trained using random oversampling to predict inpatient bed days. A. Predicting ≥7 inpatient bed days in subsequent year. B. Predicting ≥14 inpatient bed days in subsequent year. C. Predicting ≥30 inpatient bed days in subsequent year. AUC: area under the receiver operating characteristic curve; BART: Bayesian additive regression trees; KNN: k-nearest neighbor; MARS: multivariate adaptive regression splines; MLP: multilayer perceptron; PPV: positive predictive value. A higher-resolution image of this figure is available in Multimedia Appendix 2.

**Figure 4:**
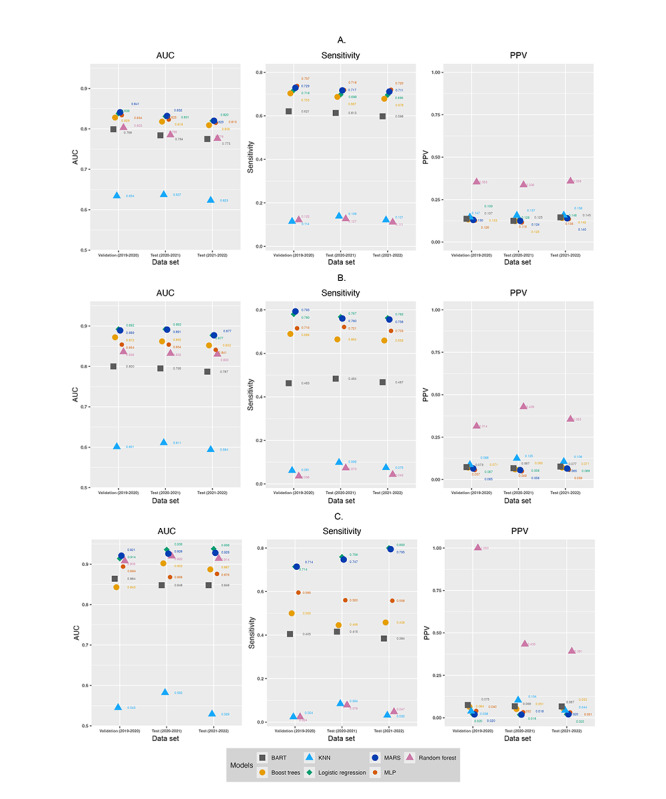
Performance of models trained using random oversampling to predict emergency department visits. A. Predicting ≥3 emergency department visits in subsequent year. B. Predicting ≥5 emergency department visits in subsequent year. C. Predicting ≥10 emergency department visits in subsequent year. A higher resolution version of this figure is available in Multimedia Appendix 2. AUC: area under the receiver operating characteristic curve; BART: Bayesian additive regression trees; KNN: k-nearest neighbor; MARS: multivariate adaptive regression splines; MLP: multilayer perceptron; PPV: positive predictive value.

As models trained with random oversampling and SMOTE-NC, where k=3 had the best AUC and sensitivity, we conducted additional analyses to evaluate their performance by testing them on 2 test data sets of 2020-2021 and 2021-2022 (Figures 3-4, Figures 3-4 in Multimedia Appendix 2, Figures S1-S2 and Tables S5-S6 in Multimedia Appendix 1). When trained with random oversampling, 4 models, that is, logistic regression, MARS, boosted trees, and MLP had consistently high AUCs across validation and test data sets. The AUC values were higher for outcomes reflecting higher utilization (ie, ≥30 inpatient bed days and ≥10 ED visits in subsequent year). These 4 models consistently had the highest sensitivity values, with sensitivity >0.65 for all outcomes except predicting ≥10 ED visits in the subsequent year. This suggests that these 4 models were able to correctly identify at least 65% of the patients with the outcome. All models, except for random forest, had similar but low PPVs across the 2 data sets.

When trained with SMOTE-NC (k=3), most models except for k-nearest neighbor and Bayesian additive regression trees models had good AUC (>0.75) across the 2 test data sets. Models had higher AUC values for outcomes reflecting higher utilization, that is, ≥30 inpatient bed days and ≥10 ED visits in the subsequent year. Compared to models trained with random oversampling, models trained with SMOTE-NC (k=3) had a wide distribution of sensitivity values, with logistic regression and MLP having similar and consistently high sensitivity values for all outcomes except predicting ≥10 ED visits in the subsequent year. Models trained with SMOTE-NC (k=3) had a wider distribution of PPV values than models trained with random oversampling.

When comparing the performance of models trained with the 2 oversampling techniques, we observed that random oversampling resulted in marginally higher AUC and sensitivity values (Figures 3-4). The narrow distribution of PPV values in models trained with random oversampling suggests that random oversampling resulted in more consistent quality of positive predictions across the best performing models.

### Explainability Analyses

From our analysis, the best performing models were logistic regression, MARS, boosted trees, and MLP that were trained with random oversampling (herein referred to as selected models). Model-specific variable importance scores for selected models except MLP were obtained; the top 10 variables are reported in Table S7 in Multimedia Appendix 1. Model-specific variable importance scores for MLP were not available through the *vip* package. Regarding the prediction of subsequent year inpatient bed days (≥7, ≥14, ≥30), age, number of ED visits (present year), CKD stages 4 and 5, and present year inpatient utilization were the most important variables. For boosted tree and MARS, the number of ED visits (present year), CKD stage, and age were the most important variables. Regarding the prediction of subsequent year ED visits, the number of ED visits (present year), CKD stage 4 and 5, mean hemoglobin A_1c_ (HbA_1c_) values, and age were the most important variables for all models. Interestingly, the number of ED visits (present year) was consistently the most important variable for all the models.

We also obtained permutation feature importance plots for selected models (Figures S3-S4 in Multimedia Appendix 1). Regarding the prediction of subsequent year inpatient bed days (≥7, ≥14, ≥30), the permutation feature importance plots corroborated the model-specific variable importance scores, indicating that age, number of ED visits (present year), CKD stage, and present year inpatient utilization were the most important variables. Interestingly, diabetes mellitus medication category was more important in predicting ≥30 inpatient bed days in the subsequent year. Regarding the prediction of subsequent year ED visits, the number of ED visits (present year) was the dominant variable for all models. Other important variables included age, CKD stage, and present year inpatient utilization.

PDPs for the 8 most important variables across selected models are illustrated in Multimedia Appendix 1. Regarding the prediction of inpatient bed days (Figures S5-S7 in Multimedia Appendix 1), the average prediction of outcomes increased steadily with age for all models. For present-year ED visits, all models demonstrated a sharp increase in average prediction from 0 to 20 visits, with a plateau close to 1.0 (for average prediction) after 20 visits. For present-year inpatient bed days, the average prediction increased with more bed days, peaking at 14-29 days for all models except MARS. For mean HbA_1c_ values, the average prediction increased with higher HbA_1c_ levels, although a U-shaped relationship was observed for MARS, boosted trees, and MLP, with the lowest average predictions around HbA_1c_ levels of 6%-7%. Regarding diabetes medication categories, patients on insulin only and those on both oral diabetic medications and insulin had higher average predictions than those on oral medications only or no medications. PDPs for selected models showed that more advanced CKD stages (CKD stage 4 and stage 5) had higher average predictions. In most models, patients with ischemic heart disease or peripheral artery disease also had higher average predictions.

Regarding the prediction of ED visits ≥3 and ≥5 times (Figures S8-S9 in Multimedia Appendix 1), the selected models showed similar observations for age, present year ED visits, mean HbA_1c_, diabetes medication categories, ischemic heart disease, and peripheral artery disease. It is noteworthy that present-year inpatient bed days did not significantly affect the predicted probability of these outcomes. For the prediction of ED visits ≥10 (Figure S10 in Multimedia Appendix 1), the PDPs aligned with the findings from both feature importance methods where the number of present year ED visits had the largest influence on average predictions, while other variables had smaller influence on average predictions.

## Discussion

### Principal Findings

In this study, we developed machine learning models to predict future inpatient and ED utilization by using sociodemographic characteristics, health indicators, diabetes-related complications, and prior utilization data from a chronic disease registry. We detailed a systematic approach to building, validating, and testing the models. Using this approach, we noted that imbalanced data distribution significantly affected model performance, often resulting in low sensitivity despite acceptable AUC values. This finding highlights the importance of considering multiple metrics, including AUC, sensitivity (recall), and PPV (precision), during model selection. We found that improved model performance can be achieved by addressing imbalanced data distribution through oversampling. We observed that random oversampling resulted in better model performance than SMOTE. Among the models trained with random oversampling, logistic regression, MARS, boosted trees, and MLP models had the best performance. Additionally, explainability analyses provided insights into how the best performing models made predictions and showed that their learned patterns were consistent with real-world knowledge, thereby supporting the validity of the models.

### Predicting Future Inpatient Bed Days and ED Visits

In our study, we used inpatient bed days and ED visits within a calendar year as service level indicators of high health care utilization. Service level utilization is important because our prior research demonstrated a rising trend in diabetes-related complications [12] and our country is experiencing persistent bed shortages and crowded EDs [26]. In this context, service level utilization indicators are useful to inform health intervention programs to ease the bed crunch and overcrowded EDs. First, patients predicted to have very high level of health care utilization (ie, inpatient bed days ≥30 or ED visits ≥10) could be candidates for intensive case management to identify potential causes for prolonged admissions or frequent ED visits. Second, patients predicted to have moderately high level of health care utilization (ie, inpatient bed days ≥14 and <30 and ED visits ≥5 and <10) could be candidates for multidisciplinary (medical and social) diabetes care programs to reduce future utilization. Finally, patients with mildly elevated health care utilization (ie, inpatient bed days ≥7 and <14 and ED visits ≥3 and <5) could be candidates for novel care models that leverage technological solutions such as the Mobile Inpatient Care at Home [27].

### Addressing Imbalanced Data Distribution by Using Data Sampling Approaches

Our study highlights the importance of addressing imbalanced data when developing machine learning models for health care applications. We observed that class imbalance can lead to acceptable AUC but low sensitivity—a phenomenon also noted in related literature [28]. Our study evaluates 2 different oversampling techniques: random oversampling and SMOTE. When comparing random oversampling with the 2 iterations of SMOTE, we found that random oversampling performed better than SMOTE (k=3), which in turn performed better than SMOTE (k=5). This could suggest that predictive models perform better when the synthetic minority class used for training is similar to the actual training data. Random oversampling duplicates existing instances, whereas SMOTE (k=3) and SMOTE (k=5) create a new synthetic minority class by interpolating between 3 and 5 closely related minority class instances, respectively. It is recognized that with oversampling techniques, models may overfit and perform poorly in other data sets [14]. To investigate this, we tested our models on 2 additional test data sets (years 2020-2021 and 2021-2022) and found no degradation in model performance. Our conclusions were that because the training data were sufficiently large, it had good quality and variety to avoid overfitting.

### Machine Learning Model Performance

Among the 7 machine learning models we tested, logistic regression, MARS, boosted trees, and MLP showed promising performance in predicting LOS across all 3 thresholds. For predicting ≥5 and ≥10 ED visits in the subsequent year, MARS and logistic regression outperformed the other models. Interestingly, logistic regression was found to be as effective as or even superior to other machine learning models in predicting health care utilization. These findings are noteworthy because while some studies have shown machine learning models to outperform traditional regression models in predicting health care utilization [3,28], others have found that machine learning models offered only limited improvement over traditional logistic regression [29]. When analyzing the model-specific variable importance scores and permutation feature importance plots for the selected models, we observed differences in the rankings of the important variables between models. However, the top 5 variables were generally consistent across selected models (Table S7 and Figures S3-S4 in Multimedia Appendix 1). In predicting inpatient LOS at all 3 thresholds, age, number of ED visits (present year), CKD stage, and inpatient bed days were the top 5 most important variables across all models. For predicting ED visits at all thresholds, the number of ED visits (present year), CKD stage, age, and mean HbA_1c_ values were the top 5 variables.

Additionally, explainability analyses using PDPs confirm what is known about high health care utilizers. Age, prior utilization in terms of ED visits and inpatient stays, and the presence of comorbidities and diabetes-related complications such as advanced stages of CKD, ischemic heart disease, and peripheral artery disease are associated with increased health care utilization. These findings suggest that current utilization is an important predictor of future utilization—a conclusion supported by similar studies [4,28]. Additionally, kidney disease has emerged as a significant predictor for future health care utilization in our cohort of patients with diabetes, as demonstrated in a recent study involving patients from the same population [30].

Interestingly, the U-shaped relationship between average prediction and HbA_1c_ values seen in many of the PDPs suggest that tight glycemic control (HbA_1c_<6%) and relaxed glycemic control (HbA_1c_≥8%) are associated with increased health care utilization. This is an interesting finding because we documented a similar U-shaped relationship previously between HbA_1c_ and incidence of diabetes mellitus–related complications in the SDR [23]. Incident complications are expected to result in ED visits or admissions. Taken together, our explainability analyses suggest that the learned patterns are consistent with real-world knowledge and therefore lend support to the validity of the model.

### Study Strengths, Limitations, and Future Research

Our study’s strengths include the use of a large multiethnic cohort and easily obtainable predictors with minimal missing data. By utilizing different thresholds of inpatient bed days and ED visits as model outcomes, our approach allows policy makers and program planners to target interventions based on the predicted need. Other practitioners intending to build predictive models for population health programs could consider a similar systematic approach to building, validating, testing, and understanding the models. Through this approach, we were able to mitigate the problems associated with class imbalance by exploring the outcomes of the 2 data sampling methods. We also validated the models across different time frames and demonstrated their validity on unseen data. Finally, our explainability analyses provided reassurance that the models were making prediction based on learned patterns consistent with real-world knowledge. However, the absence of financial data and the nonexploration of other class imbalance methods such as feature selection are key limitations that could be addressed in future studies. Our test data sets spanned the COVID-19 pandemic, a period that may have affected health-seeking behavior and health care utilization. However, the consistency of our results with those from the validation data set, which was less affected by the pandemic, suggests that these potential anomalies did not significantly impact our findings. Another potential limitation is the exclusion of patients with missing data. In the context of this study, these patients are likely to be those who are well and had minimal interaction with the health system within that year. Given the large size of the data set for this study and the significant class imbalance for patients without any of the outcomes, it is likely that excluding patients due to missing data had minimal impact on model performance.

Although our study shortlisted 4 machine learning models with similar performance across different outcomes, it remains unclear which model is the most optimal. Beyond the performance variables, we considered the confusion matrix for each of the models and observed that these models describe alternative courses of action, each with a different cost and benefit attached; we will explore this in future research. Although we have described how the results from the models can be used in practice, we acknowledge the need for a more integrated approach to model selection and decision-making criteria. In this regard, we are currently exploring additional methods to address this, specifically focusing on how to combine the outputs of the binary classification models into a single more comprehensive multiclass prediction model. To achieve this, we are investigating the use of hierarchical decision models and ensemble model approaches. These methods would allow us to integrate the predictions from individual binary models into a unified multiclass model, making it more applicable in real-world scenarios. However, these additional methods and their applications will be detailed in a follow-up study. Relatedly, the models that we developed are predictive and they are unable to provide prescriptive insights. Additional tools will be needed to be developed to profile patients and identify the most appropriate interventions for them. Finally, since our study uses data from a public regional health database in Singapore, the findings may not be generalizable to other contexts.

### Conclusion

We were able to apply common machine learning algorithms to predict future health care utilization by using inpatient bed days and ED utilization as the predicted outcomes. These predictive models will be useful to policy makers and program planners as they develop population health initiatives to improve care for patients with diabetes and manage acute health care utilization.

## Appendix

Multimedia Appendix 1 Supplementary data.

Multimedia Appendix 2 High-resolution images of Figures 1-4.

## Abbreviations

AUC: area under the receiver operating characteristic curve
CKD: chronic kidney disease
ED: emergency department
HbA_1c_: hemoglobin A_1c_
HNHC: high-need, high-cost
LOS: length of stay
MARS: multivariate adaptive regression splines
MLP: multilayer perceptron
PDP: partial dependence plot
PPV: positive predictive value
SDR: SingHealth Diabetes Registry
SingHealth: Singapore Health Services
SMOTE-NC: synthetic minority oversampling technique-nominal continuous

## Data Availability

Data cannot be shared publicly because of Singapore Health Services Cluster Data Policy on data sharing restrictions. Data are available from the Singapore Health Services Diabetes Registry Committee for researchers who meet the criteria for access to confidential data. The criteria include institutional review board approval, data use approval, and a research collaboration agreement. The point of contact for the Singapore Health Services Diabetes Registry Disease Registry Committee is Ms Lee Thong Shuen (email: lee.thong.shuen@singhealth.com.sg).
