# Supplementary material for: Machine Learning–Based Prediction for High Health Care Utilizers by Using a Multi-Institutional Diabetes Registry: Model Training and Evaluation
Source: JMIR AI. 2024 Oct 17;3:e58463. doi: 10.2196/58463 (PMC11528163; doi:10.2196/58463)

**Multimedia Appendix 1**

**Contents**

**Supplementary Tables**

**Table S1. Detailed criteria used for ascertaining diabetes-related complications.**

**Table S2. Variables utilized in model building.**

**Table S3. Consolidated reporting guidelines for prognostic and diagnostic machine learning modeling studies.**

**Table S4. Performance of various models by over-sampling technique and outcomes.**

**Table S5. Model performance in validation and test datasets for models trained with random over-sampling.**

**Table S6. Model performance in validation and test datasets for models trained with SMOTE (k = 3).**

**Table S7. Top 10 variable importance score for selected models by outcomes.**

**Supplementary Figures**

**Figure S1. Performance of models trained using SMOTE-NC (K=3) to predict inpatient bed days.**

**Figure S2. Performance of models trained using SMOTE-NC (k=3) to predict emergency department visits.**

**Figure S3. Permutation feature importance plots for models trained using random over-sampling to predict inpatient bed days.**

**Figure S4. Permutation feature importance plots for models trained using random over-sampling to predict emergency department visits.**

**Figure S5. Partial dependence plots for selected variables for models trained using random over-sampling to predict inpatient bed days ≥ 7 days.**

**Figure S6. Partial dependence plots for selected variables for models trained using random over-sampling to predict inpatient bed days ≥ 14 days.**

**Figure S7. Partial dependence plots for selected variables for models trained using random over-sampling to predict inpatient bed days ≥ 30 days.**

**Figure S8. Partial dependence plots for selected variables for models trained using random over-sampling to predict emergency department visits ≥ 3 visits.**

**Figure S9. Partial dependence plots for selected variables for models trained using random over-sampling to predict emergency department visits ≥ 5 visits.**

**Figure S10. Partial dependence plots for selected variables for models trained using random over-sampling to predict emergency department visits ≥ 10 visits.**

**Table S1. Detailed criteria used for ascertaining diabetes-related complications.**

| Outcome | Criterion | | | Healthcare setting |
| --- | --- | --- | --- | --- |
|  | ICD-10 codes | SNOMED codes | SHPWKC or Surgical codes |  |
| Macrovascular complications | | | | |
| Ischemic heart disease | ICD-10 I20.x, I21.x, I22.x, I24.x, I25.x | 89331010, 299761018, 299762013, 299782012, 299783019, 299784013, 350346019, 459859010, 1040100013, 1051100013, 1210398011, 1210399015, 1210505015, 2534663012, 2534664018, 2534667013, 2534671011, 2534672016, 2535853014, 2535854015, 2535923015, 2536394018, 2536395017, 2536397013, 2536398015, 2537479013, 2537480011, 2842265012, 2951007019, 2951008012, 2951009016, 2951010014, 2986368010, 2986970011, 3289323011 | 042A (Chronic ischemic heart disease), 042B (Acute ischemic heart disease), | Inpatient, outpatient and primary care. |
| Acute myocardial infarction | ICD-10 I21.x, I22.x, I 25.2 | - | - | Inpatient |
| Peripheral arterial disease | ICD-10 I70.2, I73.1, I73.8-73.9 | 18413012, 23786012, 46785011, 87530017, 105536013, 205224017, 300607015, 350531010, 350534019, 350535018, 350536017, 350543011, 350544017, 350545016, 350546015, 350547012, 350548019, 357894012, 357896014, 357897017, 357898010, 411512011, 443199013, 451376017, 456609017, 456610010, 456617013, 458506014, 458507017, 473473019, 484263016, 1232467011, 1233009011, 1779239017, 1779317016, 1787049010, 1787050010, 1787052019, 2157354010, 2162392019, 2693498010, 2693575017, 2693576016, 2773672016, 2921338010, 2923092011, 2923094012, 2923309015, 2923379016, 2955895013, 2956118016, 2983747016, 2983776018, 2985567012, 3040857018, 3289284017 | 047B (PVD), surgical codes (SD720A (Artery, Stenosis / Occlusion, Percutaneous Transluminal Angioplasty (PTA), Difficult (Eg Subintimal PTA, Below Knee PTA)), SD728A (Artery, Various Lesions, Endovascular Stent Placement), SD713A (Artery, Bypass, Above/Below-Knee With Vein), SD717A (Artery, Femoral Artery Angioplasty), SD721A (Artery, Stenosis / Occlusion, Percutaneous Transluminal Angioplasty (Pta), Simple), SD714A (Artery, Bypass, Distal Leg/Pedal With Vein), SD719A (Artery, Stenosis / Occlusion, Percutaneous Atherectomy (Mechanical Or Laser))) | Inpatient, outpatient and primary care. |
| Major or minor lower extremity amputation | - | - | Mapped from surgical codes (SB010L, SB400T, SB401T, SB707T, SB708T, SB809L, SB829T, SB830T.) |  |
| Diabetic foot and peripheral angiopathy | ICD-10 E10.51-10.52, E10.73, E11.51-11.52, E11.73, E13.73, E14.51-14.52, E14.73 | 286569015, 286570019, 300505017, 300506016, 308360012, 309178017, 357897017, 357898010, 357902016, 417658010, 451404016, 451405015, 498448012, 512081010, 1209795012, 1488395018, 1488396017, 1778315019, 1781954013, 2532976019, 2532977011, 2533104014, 2621545012, 2621551019, 2621797015, 2621798013, 2987211019, 3040857018 | 018K (DM Foot) | Inpatient, outpatient and primary care. |
| Stroke | ICD-10 I60.0-61.9, I62.9, I69.0-69.2, I63.x, I64.x, I69.3 – I69.4, G46.0-46.8 | 3421016, 13756016, 36011016, 39067013, 39068015, 42129011, 49074018, 79273012, 96391014, 123408011, 124627016, 124628014, 124629018, 134436018, 158109012, 158113017, 158114011, 158118014, 178528011, 251701010, 300271012, 300272017, 300277011, 300279014, 300321011, 300322016, 300365015, 300366019, 300369014, 300408017, 345635016, 345636015, 345637012, 345638019, 345640012, 345641011, 345642016, 345646018, 345647010, 345649013, 345651012, 345652017, 345653010, 345656019, 345659014, 345667018, 345671015, 345671015, 345675012, 345676013, 345677016, 345682011, 345694019, 345696017, 405377010, 405379013, 409859018, 409860011, 412270012, 412997011, 419212013, 419213015, 444898010, 450609012, 451133011, 451134017, 451371010, 475553012, 481028017, 481580017, 496232015, 502878012, 503469016, 1209750017, 1209751018, 1212072018, 1216125018, 1217312010, 1217630014, 1218800017, 1229580019, 2474651019, 2474986019, 2475187018, 2476091017, 2535208016, 2644233012, 2644234018, 2674093010, 2674127019, 2675250011, 2694009014, 2695743015, 2770034014, 2772089016, 2818561018, 2819959010, 2819960017, 2901452016, 2901453014, 2914970017, 2916313015, 2920376012, 2966556014, 2966565019, 2966596011, 2966602014, 2966650013, 2967471017, 2967506011, 2967537011, 2967598015, 2967601013, 2967609010, 2967628015, 2967650019, 2967701015, 2981993016, 2984150019, 2984188017, 2984239014, 2986393012, 2986825016, 2986886017, 2987976012, 3023367013, 3023374015, 3037871010, | 046A (Stroke (Haemorrhage)), 046C (Stroke (Ischaemic/infarct)), 046B (Stroke (Not specified) | Inpatient, outpatient and primary care. |
| Microvascular complications | | | | |
| Ophthalmopathy | ICD-10 E10.31, E10.39, E11.31-11.39, E14.31-14.39, H330, H332, H334, H342, H348, H350, H352, H353, H354, H358, H431 | 9093013, 33037011, 42066015, 42768011, 52406015, 70200018, 89378011, 93460014, 98476015, 158593013, 251652015, 297723010, 297745011, 297802010, 297803017, 297806013, 297807016, 347642015, 347645018, 347650012, 347651011, 347657010, 347660015, 347720013, 347721012, 368990015, 368991016, 456707011, 456712012, 456713019, 456714013, 456715014, 456716010, 456717018, 484848010, 486663012, 486665017, 493192015, 1228202019, 1228203012, 1230610015, 1231068014, 1484867016, 1488434016, 1773466014, 1774748012, 1774750016, 1775724010, 1775725011, 1779161016, 1779166014, 1783717018, 1783897011, 1784103011, 1785151013, 1785152018, 1785155016, 1785158019, 1785161018, 1785332013, 2164671014, 2164673012, 2551706016, 2579342012, 2579343019, 2579521014, 2618238018, 2618241010, 2620592018, 2620593011, 2621399015, 2621472018, 2621576018, 2642672017, 2643005014, 2921114017, 2967698011, 2967760017, 2967787018, 2989730012, 2990445013, 3004798011, 3013241019, 3303450013, | 018Q (DM Retinopathy) | Inpatient, outpatient and primary care. |
| Nephropathy | Criteria: eGFR <60mL/min/1.73m^2^ (most recent) and/or urine albumin-creatinine ratio (uACR) ≥ 30mg/g and/or urine protein/creatinine ratio ≥ 0.20 g/g. Laboratory information system data and CKD-EPI formula used to calculate eGFR. | | |  |
| Neuropathy | ICD-10 E10.40-10.44, E11.40-11.44, E14.40-14.43, G629 | 299011, 8504012, 23368011, 34691019, 59668014, 65526011, 65642012, 70659018, 82373015, 84344019, 94571018, 94572013, 100945019, 100951012, 131983013, 135747015, 141590010, 158470017, 158471018, 170583016, 194672015, 195776019, 206412019, 255456017, 255459012, 255462010, 255465012, 297550019, 297551015, 297552010, 323630017, 345486016, 345487013, 345488015, 345489011, 345490019, 345491015, 345492010, 345524019, 369417015, 413629013, 414606010, 417464011, 473862018, 475273015, 477766010, 478654013, 480783010, 480785015, 480788018, 502316014, 1230890017, 1480220018, 1483041015, 1493575013, 1777429019, 1778546016, 1786560016, 1786593013, 1786596017, 1786598016, 2645902011, 2667589016, 2667594016, 2694146010, 2870944013, 2920418018, 2967619016, 2967625011, 2967635011, 2967939019, 3005273015, 3286275018, 3286275018, 3297353013 | 018P (DM Neuropathy) | Inpatient, outpatient and primary care. |

**Table S2. Variables utilized in model building.**

| No. | Variables | Description of variable |
| --- | --- | --- |
| 1 | Age | Continuous |
| 2 | Gender | Dichotomous (Male, female) |
| 3 | Ethnicity | Categorical (Chinese, Malay, Indian, other) |
| 4 | Housing type | Categorical (One and two-room flat, three-room flat, four-room flat, five-room flat, private condominium, private landed property) |
| 5 | Rental block | Dichotomous (Absent, present) |
| 6 | Singapore Housing Index | Continuous |
| 7 | Hypertension | Dichotomous (Absent, present) |
| 8 | Hyperlipidemia | Dichotomous (Absent, present) |
| 9 | Mean Hba1c | Continuous |
| 10 | DM meds category | Categorical (None, oral only, insulin only, oral and insulin) |
| 11 | CKD stage | Categorical (Stage 1, stage 2, stage 3A, stage 3B, stage 4, stage 5) |
| 12 | Dialysis | Dichotomous (Absent, present) |
| 13 | IHD | Dichotomous (Absent, present) |
| 14 | Peripheral arterial disease | Dichotomous (Absent, present) |
| 15 | Stroke (hemorrhagic) | Dichotomous (Absent, present) |
| 16 | Stroke (ischemic) | Dichotomous (Absent, present) |
| 17 | Major lower extremity amputation (LEA) | Dichotomous (Absent, present) |
| 18 | Minor lower extremity amputation (LEA) | Dichotomous (Absent, present) |
| 19 | Diabetic foot | Dichotomous (Absent, present) |
| 20 | Diabetic eye complications | Dichotomous (Absent, present) |
| 21 | Nephropathy | Dichotomous (Absent, present) |
| 22 | Neuropathy | Dichotomous (Absent, present) |
| 23 | Present year inpatient bed days | Categorical (0 days, 1-2 days, 3-6 days, 7-13 days, 14-29 days, ≥ 30 days) |
| 24 | Present year number of emergency department visits | Interval |

**Table S3. Consolidated reporting guidelines for prognostic and diagnostic machine learning modeling studies.**

| **#** | **Item** | **Y** | **N** | **NA** | **Location / Reasoning** |
| --- | --- | --- | --- | --- | --- |
| **Study Details** | | | | | |
| 1.1 | *The medical/clinical task of interest* | ✓ |  |  | Pg 5; lines 78-82 |
| 1.2 | *The research question* | ✓ |  |  | Pg 5; lines 84-88 |
| 1.3 | *Current medical/clinical practice* | ✓ |  |  | Pg 4; lines 53-63 Pg 5; lines 84-86 |
| 1.4 | *The known predictors and confounders to what is being predicted / diagnosed* | ✓ |  |  | Pg 7; lines 126-133 |
| 1.5 | *The overall study design* | ✓ |  |  | Pg 7-9; lines 143-184 |
| 1.6 | *The medical institutional setting(s)* | ✓ |  |  | Pg 5-6; lines 93-98 |
| 1.7 | *The target patient population* | ✓ |  |  | Pg 5-6; lines 93-98 |
| 1.8 | *The intended use of the ML model* | ✓ |  |  | Pg 5; lines 88-89 |
| 1.9 | *Existing model performance benchmarks for this task* |  |  | ✓ | There are no existing model benchmarks for this task, see Pg 5; lines 85-86 |
| 1.10 | *Ethical and other regulatory approvals obtained* | ✓ |  |  | Pg 9-10; lines 190-192 |
| **The Data** | | | | | |
| 2.1 | *Inclusion / exclusion criteria for the patient cohort* | ✓ |  |  | Pg 7; lines 136-140 |
| 2.2 | *Methods of data collection* | ✓ |  |  | Pg 5; lines 93-94. Data from a registry was used. Data collection was previously described. |
| 2.3 | *Bias introduced due to the method of data collection used* | ✓ |  |  | Pg 23; lines 434-439. Data from the registry spanned the continuum of care (primary to tertiary) and is reasonable to expect the data to be comprehensive since the registry is well established. We did not describe the biases upfront, but discussed it in the limitations section of the discussion. |
| 2.4 | *Data characteristics* | ✓ |  |  | Page 10-15; lines 199-232 |
| 2.5 | *Methods of data transformations and preprocessing applied* | ✓ |  |  | Page 8; lines 142-155 |
| 2.6 | *Known quality issues with the data* | ✓ |  |  | Page 10-11; lines 199-204. We used complete case analysis and described the extent of missing data in Table 1. |
| 2.7 | *Sample size calculation* |  |  | ✓ | The whole registry was used, no sample size calculation was necessary. |
| 2.8 | *Data Availability* | ✓ |  |  | Page 30-31; lines 496-502. The data availability statement is provided in the acknowledgements section. |
| **Methodology** | | | | | |
| 3.1 | *Strategies for handling missing data* |  |  | ✓ | Complete case analysis was used, the dataset was large and missing data is unlikely to have impacted the model training as described in Page 27-28; lines 446-450. |
| 3.2 | *Strategies for addressing class imbalance* | ✓ |  |  | Page 8; lines 142-155. |
| 3.3 | *Strategies for reducing dimensionality of data* | ✓ |  |  | Page 7; lines 129-133. We utilized variables (features) that were both readily available and clinically relevant. This was done to reduce the amount of missing data that would be excluded. |
| 3.4 | *Strategies for handling outliers* |  |  | ✓ | Our study is interested in identifying patient who were high utilizers. These patients are inherently outliers. |
| 3.5 | *Strategies for data augmentation* |  | ✓ |  | Data augmentation was not required as the training dataset was large. |
| 3.6 | *Strategies for model pre-training* |  | ✓ |  | We did not perform model pre-training, only model training. |
| 3.7 | *The rationale for selecting the machine learning algorithm* |  | ✓ |  | We wanted to explore the performance of as many models as possible and described our selection process throughout the manuscript. |
| 3.8 | *The method of evaluating model performance during training* | ✓ |  |  | Page 8-9; lines 157-165. |
| 3.9 | *The method used for hyperparameter tuning* |  | ✓ |  | No hyperparameter tuning was performed, as the intent of the study was to build baseline models to understand the problem and dataset whilst prioritizing model simplicity and interpretability (see Page 9; lines 173-175). |
| 3.10 | *Model’s output adjustments* |  | ✓ |  | No output adjustments was performed. |
| **Evaluation** | | | | | |
| 4.1 | *Performance metrics used to evaluate the model* | ✓ |  |  | Page 8; lines 157-165. |
| 4.2 | *The cost or consequence of errors* |  | ✓ |  | Cost and consequences of the error was not explored in this study. We are currently working on a future study to evaluate the financial costs and consequences of the best performing models before deploying them. |
| 4.3 | *The results of internal validation* | ✓ |  |  | Page 16-17; lines 249-253. Additionally, we provide detailed information of the models’ performance in the Multimedia Appendix. |
| 4.4 | *The final model hyperparameters* |  | ✓ |  | Hyperparameter tuning was not performed |
| 4.5 | *Model evaluation on an external dataset* | ✓ |  |  | Page 18-19; lines 255-259. Additionally, we provide detailed information of the models’ performance in the Multimedia Appendix. We describe the models’ performance on unseen datasets from subsequent years. |
| 4.6 | *Characteristics relevant for detecting data shift and drift* | ✓ |  |  | Page 11-13; Table 1. The characteristics of the training and tests datasets are detailed. No significant data shift and drift was noted. |
| **Explainability and Transparency** | | | | | |
| 5.1 | *The most important features and how they relate to the outcome(s)* | ✓ |  |  | Page 21-23; lines 291-339. |
| 5.2 | *Plausibility of model outputs* | ✓ |  |  | Page 22-23; lines 316-339, and Page 26; lines 401-426. |
| 5.3 | *Interpretation of model's results by an end-user* | ✓ |  |  | Page 24; lines 357-372. |

**Table S4. Performance of various models by over-sampling technique and outcomes.**

| **Predicting next year stay >= 7 days** | | | | | | | | | | | | |
| --- | --- | --- | --- | --- | --- | --- | --- | --- | --- | --- | --- | --- |
| **Over-sampling Technique** | **SMOTE-NC (k = 5)** | | | **SMOTE-NC (k = 3)** | | | **UpSample (random)** | | | **No over-sampling** | | |
|  | Model results (AUC) | Sensitivity | Positive predictive value | Model results (AUC) | Sensitivity | Positive predictive value | Model results (AUC) | Sensitivity | Positive predictive value | Model results (AUC) | Sensitivity | Positive predictive value |
| Logistic regression | 0.762 | 0.632 | 0.196 | 0.777 | 0.651 | 0.200 | 0.807 | 0.675 | 0.222 | 0.803 | 0.101 | 0.527 |
| Random forest | 0.771 | 0.364 | 0.282 | 0.776 | 0.357 | 0.296 | 0.785 | 0.261 | 0.358 | 0.806 | 0.072 | 0.630 |
| Boost trees | 0.773 | 0.436 | 0.264 | 0.774 | 0.431 | 0.270 | 0.806 | 0.694 | 0.212 | 0.809 | 0.094 | 0.598 |
| MLP | 0.757 | 0.621 | 0.196 | 0.777 | 0.640 | 0.202 | 0.806 | 0.709 | 0.207 | 0.807 | 0.103 | 0.567 |
| MARS | 0.760 | 0.463 | 0.269 | 0.766 | 0.460 | 0.267 | 0.806 | 0.687 | 0.215 | 0.798 | 0.100 | 0.516 |
| KNN | 0.665 | 0.364 | 0.153 | 0.661 | 0.320 | 0.151 | 0.653 | 0.196 | 0.213 | 0.665 | 0.199 | 0.212 |
| BART | 0.733 | 0.508 | 0.198 | 0.745 | 0.517 | 0.199 | 0.794 | 0.673 | 0.207 | 0.806 | 0.082 | 0.595 |
|  | | | | | | | | | | | | |
| **Predicting next year stay >= 14 days** | | | | | | | | | | | | |
| **Over-sampling Technique** | **SMOTE-NC (k = 5)** | | | **SMOTE-NC (k = 3)** | | | **UpSample (random)** | | | **No over-sampling** | | |
|  | Model results (AUC) | Sensitivity | Positive predictive value | Model results (AUC) | Sensitivity | Positive predictive value | Model results (AUC) | Sensitivity | Positive predictive value | Model results (AUC) | Sensitivity | Positive predictive value |
| Logistic regression | 0.769 | 0.626 | 0.129 | 0.79 | 0.656 | 0.136 | 0.827 | 0.702 | 0.155 | 0.822 | 0.064 | 0.491 |
| Random forest | 0.780 | 0.302 | 0.195 | 0.786 | 0.285 | 0.221 | 0.799 | 0.136 | 0.327 | 0.827 | 0.025 | 0.477 |
| Boost trees | 0.783 | 0.438 | 0.191 | 0.792 | 0.458 | 0.208 | 0.831 | 0.730 | 0.149 | 0.830 | 0.044 | 0.509 |
| MLP | 0.765 | 0.602 | 0.130 | 0.782 | 0.635 | 0.132 | 0.827 | 0.727 | 0.140 | 0.828 | 0.000 | NaN |
| MARS | 0.752 | 0.425 | 0.172 | 0.771 | 0.470 | 0.192 | 0.828 | 0.733 | 0.155 | 0.816 | 0.067 | 0.513 |
| KNN | 0.674 | 0.284 | 0.103 | 0.667 | 0.268 | 0.111 | 0.639 | 0.131 | 0.150 | 0.660 | 0.155 | 0.166 |
| BART | 0.735 | 0.469 | 0.133 | 0.737 | 0.473 | 0.129 | 0.805 | 0.652 | 0.145 | 0.827 | 0.038 | 0.540 |
|  | | | | | | | | | | | | |
| **Predicting next year stay >= 30 days** | | | | | | | | | | | | |
| **Over-sampling Technique** | **SMOTE-NC (k = 5)** | | | **SMOTE-NC (k = 3)** | | | **UpSample (random)** | | | **No over-sampling** | | |
|  | Model results (AUC) | Sensitivity | Positive predictive value | Model results (AUC) | Sensitivity | Positive predictive value | Model results (AUC) | Sensitivity | Positive predictive value | Model results (AUC) | Sensitivity | Positive predictive value |
| Logistic regression | 0.779 | 0.606 | 0.065 | 0.804 | 0.657 | 0.069 | 0.849 | 0.724 | 0.081 | 0.845 | 0.045 | 0.615 |
| Random forest | 0.786 | 0.175 | 0.114 | 0.800 | 0.159 | 0.131 | 0.809 | 0.054 | 0.333 | 0.846 | 0.009 | 0.455 |
| Boost trees | 0.796 | 0.407 | 0.110 | 0.805 | 0.442 | 0.116 | 0.840 | 0.700 | 0.082 | 0.849 | 0.030 | 0.516 |
| MLP | 0.773 | 0.575 | 0.070 | 0.792 | 0.657 | 0.069 | 0.835 | 0.729 | 0.075 | 0.845 | 0.000 | NaN |
| MARS | 0.754 | 0.418 | 0.093 | 0.788 | 0.463 | 0.100 | 0.852 | 0.748 | 0.082 | 0.815 | 0.060 | 0.492 |
| KNN | 0.652 | 0.192 | 0.058 | 0.652 | 0.181 | 0.067 | 0.603 | 0.078 | 0.093 | 0.614 | 0.080 | 0.083 |
| BART | 0.723 | 0.377 | 0.064 | 0.729 | 0.410 | 0.072 | 0.792 | 0.539 | 0.081 | 0.843 | 0.022 | 0.706 |
|  | | | | | | | | | | | | |
| **Predicting next ED visit >= 3** | | | | | | | | | | | | |
| **Over-sampling Technique** | **SMOTE-NC (k = 5)** | | | **SMOTE-NC (k = 3)** | | | **UpSample (random)** | | | **No over-sampling** | | |
|  | Model results (AUC) | Sensitivity | Positive predictive value | Model results (AUC) | Sensitivity | Positive predictive value | Model results (AUC) | Sensitivity | Positive predictive value | Model results (AUC) | Sensitivity | Positive predictive value |
| Logistic regression | 0.775 | 0.613 | 0.128 | 0.800 | 0.648 | 0.133 | 0.838 | 0.719 | 0.140 | 0.833 | 0.076 | 0.525 |
| Random forest | 0.781 | 0.291 | 0.214 | 0.793 | 0.279 | 0.230 | 0.802 | 0.125 | 0.364 | 0.832 | 0.040 | 0.661 |
| Boost trees | 0.797 | 0.438 | 0.221 | 0.806 | 0.460 | 0.222 | 0.830 | 0.685 | 0.134 | 0.837 | 0.060 | 0.630 |
| MLP | 0.777 | 0.647 | 0.112 | 0.792 | 0.642 | 0.118 | 0.832 | 0.728 | 0.130 | 0.837 | 0.066 | 0.500 |
| MARS | 0.751 | 0.411 | 0.191 | 0.787 | 0.455 | 0.207 | 0.841 | 0.732 | 0.131 | 0.825 | 0.075 | 0.593 |
| KNN | 0.666 | 0.270 | 0.090 | 0.663 | 0.254 | 0.099 | 0.634 | 0.113 | 0.146 | 0.662 | 0.145 | 0.161 |
| BART | 0.704 | 0.439 | 0.121 | 0.708 | 0.460 | 0.121 | 0.793 | 0.612 | 0.135 | 0.823 | 0.059 | 0.600 |
|  | | | | | | | | | | | | |
| **Predicting next ED visit >= 5** | | | | | | | | | | | | |
| **Over-sampling Technique** | **SMOTE-NC (k = 5)** | | | **SMOTE-NC (k = 3)** | | | **UpSample (random)** | | | **No over-sampling** | | |
|  | Model results (AUC) | Sensitivity | Positive predictive value | Model results (AUC) | Sensitivity | Positive predictive value | Model results (AUC) | Sensitivity | Positive predictive value | Model results (AUC) | Sensitivity | Positive predictive value |
| Logistic regression | 0.831 | 0.621 | 0.060 | 0.855 | 0.686 | 0.062 | 0.892 | 0.783 | 0.067 | 0.885 | 0.091 | 0.622 |
| Random forest | 0.818 | 0.178 | 0.123 | 0.828 | 0.155 | 0.155 | 0.837 | 0.039 | 0.324 | 0.878 | 0.019 | 0.750 |
| Boost trees | 0.834 | 0.443 | 0.118 | 0.842 | 0.417 | 0.104 | 0.871 | 0.650 | 0.073 | 0.883 | 0.049 | 0.500 |
| MLP | 0.792 | 0.576 | 0.053 | 0.825 | 0.625 | 0.059 | 0.866 | 0.731 | 0.057 | 0.846 | 0.000 | NaN |
| MARS | 0.816 | 0.521 | 0.110 | 0.843 | 0.515 | 0.115 | 0.889 | 0.793 | 0.066 | 0.874 | 0.058 | 0.667 |
| KNN | 0.651 | 0.162 | 0.051 | 0.663 | 0.149 | 0.058 | 0.602 | 0.061 | 0.088 | 0.632 | 0.071 | 0.093 |
| BART | 0.702 | 0.317 | 0.054 | 0.727 | 0.353 | 0.064 | 0.812 | 0.479 | 0.074 | 0.876 | 0.049 | 0.833 |
|  | | | | | | | | | | | | |
| **Predicting next ED visit >= 10** | | | | | | | | | | | | |
| **Over-sampling Technique** | **SMOTE-NC (k = 5)** | | | **SMOTE-NC (k = 3)** | | | **UpSample (random)** | | | **No over-sampling** | | |
|  | Model results (AUC) | Sensitivity | Positive predictive value | Model results (AUC) | Sensitivity | Positive predictive value | Model results (AUC) | Sensitivity | Positive predictive value | Model results (AUC) | Sensitivity | Positive predictive value |
| Logistic regression | 0.785 | 0.524 | 0.018 | 0.869 | 0.714 | 0.022 | 0.913 | 0.714 | 0.020 | 0.932 | 0.095 | 0.444 |
| Random forest | 0.878 | 0.048 | 0.077 | 0.912 | 0.048 | 0.125 | 0.906 | 0.024 | 1.000 | 0.906 | 0.000 | NaN |
| Boost trees | 0.847 | 0.476 | 0.093 | 0.915 | 0.429 | 0.075 | 0.875 | 0.476 | 0.061 | 0.94 | 0.119 | 0.625 |
| MLP | 0.770 | 0.333 | 0.033 | 0.878 | 0.548 | 0.030 | 0.859 | 0.619 | 0.026 | 0.5 | 0.000 | NaN |
| MARS | 0.823 | 0.619 | 0.034 | 0.897 | 0.619 | 0.029 | 0.915 | 0.714 | 0.019 | 0.943 | 0.071 | 1 |
| KNN | 0.565 | 0.119 | 0.058 | 0.578 | 0.071 | 0.035 | 0.545 | 0.024 | 0.038 | 0.617 | 0.119 | 0.172 |
| BART | 0.83 | 0.333 | 0.055 | 0.815 | 0.286 | 0.050 | 0.874 | 0.476 | 0.085 | 0.926 | 0.024 | 1 |
|  | | | | | | | | | | | | |
| Remarks: * NaN: Not a number | | | | | | | | | | | | |

**Table S5. Model performance in validation and test datasets for models trained with random over-sampling.**

| **Predicting next year stay >= 7 days** | | | | | | | | | | | | | | | | | | | | | | | |
| --- | --- | --- | --- | --- | --- | --- | --- | --- | --- | --- | --- | --- | --- | --- | --- | --- | --- | --- | --- | --- | --- | --- | --- |
| **Train-validation (2019-2020)** | | | | | | | |  | **Test (2020-2021)** | | | | | | |  | **Test (2021-2022)** | | | | | | |
|  | Model results (AUC) | PR AUC | Sensitivity | Specificity | Positive predictive value | Negative predictive value | F1 |  | Model results (AUC) | PR AUC | Sensitivity | Specificity | Positive predictive value | Negative predictive value | F1 |  | Model results (AUC) | PR AUC | Sensitivity | Specificity | Positive predictive value | Negative predictive value | F1 |
| Logistic regression | 0.807 | 0.322 | 0.675 | 0.791 | 0.222 | 0.965 | 0.335 |  | 0.806 | 0.319 | 0.687 | 0.774 | 0.228 | 0.962 | 0.342 |  | 0.801 | 0.334 | 0.700 | 0.759 | 0.247 | 0.957 | 0.365 |
| Random forest | 0.786 | 0.277 | 0.262 | 0.958 | 0.356 | 0.936 | 0.302 |  | 0.781 | 0.281 | 0.251 | 0.955 | 0.349 | 0.929 | 0.292 |  | 0.782 | 0.304 | 0.272 | 0.949 | 0.377 | 0.920 | 0.316 |
| Boost trees | 0.807 | 0.323 | 0.692 | 0.766 | 0.208 | 0.966 | 0.320 |  | 0.804 | 0.319 | 0.709 | 0.749 | 0.215 | 0.964 | 0.330 |  | 0.801 | 0.341 | 0.720 | 0.738 | 0.237 | 0.959 | 0.357 |
| MLP | 0.808 | 0.307 | 0.723 | 0.750 | 0.205 | 0.968 | 0.319 |  | 0.806 | 0.316 | 0.736 | 0.735 | 0.213 | 0.966 | 0.330 |  | 0.802 | 0.336 | 0.739 | 0.725 | 0.232 | 0.961 | 0.353 |
| MARS | 0.807 | 0.317 | 0.687 | 0.778 | 0.216 | 0.965 | 0.328 |  | 0.803 | 0.310 | 0.689 | 0.772 | 0.227 | 0.962 | 0.341 |  | 0.799 | 0.327 | 0.698 | 0.756 | 0.244 | 0.957 | 0.362 |
| KNN | 0.653 | 0.239 | 0.195 | 0.935 | 0.210 | 0.929 | 0.202 |  | 0.659 | 0.256 | 0.204 | 0.934 | 0.232 | 0.924 | 0.217 |  | 0.659 | 0.272 | 0.203 | 0.931 | 0.249 | 0.912 | 0.224 |
| BART | 0.793 | 0.303 | 0.676 | 0.772 | 0.208 | 0.964 | 0.318 |  | 0.796 | 0.303 | 0.696 | 0.749 | 0.212 | 0.962 | 0.325 |  | 0.794 | 0.325 | 0.704 | 0.748 | 0.239 | 0.957 | 0.357 |
|  | | | | | | | | | | | | | | | | | | | | | | | |
| **Predicting next year stay >= 14 days** | | | | | | | | | | | | | | | | | | | | | | | |
| **Train-validation (2019-2020)** | | | | | | | |  | **Test (2020-2021)** | | | | | | |  | **Test (2021-2022)** | | | | | | |
|  | Model results (AUC) | PR AUC | Sensitivity | Specificity | Positive predictive value | Negative predictive value | F1 |  | Model results (AUC) | PR AUC | Sensitivity | Specificity | Positive predictive value | Negative predictive value | F1 |  | Model results (AUC) | PR AUC | Sensitivity | Specificity | Positive predictive value | Negative predictive value | F1 |
| Logistic regression | 0.827 | 0.254 | 0.704 | 0.803 | 0.155 | 0.981 | 0.254 |  | 0.827 | 0.259 | 0.708 | 0.788 | 0.161 | 0.979 | 0.262 |  | 0.823 | 0.265 | 0.724 | 0.770 | 0.174 | 0.977 | 0.280 |
| Random forest | 0.797 | 0.193 | 0.140 | 0.986 | 0.340 | 0.957 | 0.199 |  | 0.791 | 0.203 | 0.124 | 0.985 | 0.321 | 0.951 | 0.179 |  | 0.793 | 0.217 | 0.129 | 0.983 | 0.339 | 0.944 | 0.187 |
| Boost trees | 0.827 | 0.252 | 0.705 | 0.792 | 0.148 | 0.981 | 0.245 |  | 0.823 | 0.250 | 0.710 | 0.779 | 0.156 | 0.979 | 0.256 |  | 0.820 | 0.269 | 0.720 | 0.765 | 0.170 | 0.976 | 0.275 |
| MLP | 0.823 | 0.250 | 0.736 | 0.770 | 0.141 | 0.983 | 0.237 |  | 0.823 | 0.244 | 0.745 | 0.755 | 0.149 | 0.981 | 0.248 |  | 0.820 | 0.261 | 0.752 | 0.743 | 0.163 | 0.978 | 0.268 |
| MARS | 0.830 | 0.253 | 0.730 | 0.795 | 0.154 | 0.983 | 0.255 |  | 0.824 | 0.246 | 0.702 | 0.789 | 0.160 | 0.979 | 0.261 |  | 0.821 | 0.256 | 0.720 | 0.772 | 0.174 | 0.976 | 0.281 |
| KNN | 0.639 | 0.168 | 0.130 | 0.962 | 0.148 | 0.956 | 0.138 |  | 0.649 | 0.193 | 0.152 | 0.962 | 0.185 | 0.952 | 0.167 |  | 0.641 | 0.198 | 0.147 | 0.958 | 0.191 | 0.944 | 0.166 |
| BART | 0.806 | 0.212 | 0.649 | 0.803 | 0.145 | 0.978 | 0.237 |  | 0.805 | 0.217 | 0.670 | 0.788 | 0.154 | 0.977 | 0.250 |  | 0.804 | 0.237 | 0.678 | 0.782 | 0.172 | 0.973 | 0.275 |
|  | | | | | | | | | | | | | | | | | | | | | | | |
| **Predicting next year stay >= 14 days** | | | | | | | | | | | | | | | | | | | | | | | |
| **Train-validation (2019-2020)** | | | | | | | |  | **Test (2020-2021)** | | | | | | |  | **Test (2021-2022)** | | | | | | |
|  | Model results (AUC) | PR AUC | Sensitivity | Specificity | Positive predictive value | Negative predictive value | F1 |  | Model results (AUC) | PR AUC | Sensitivity | Specificity | Positive predictive value | Negative predictive value | F1 |  | Model results (AUC) | PR AUC | Sensitivity | Specificity | Positive predictive value | Negative predictive value | F1 |
| Logistic regression | 0.849 | 0.184 | 0.724 | 0.822 | 0.082 | 0.993 | 0.147 |  | 0.841 | 0.168 | 0.707 | 0.812 | 0.087 | 0.991 | 0.155 |  | 0.843 | 0.170 | 0.739 | 0.786 | 0.094 | 0.990 | 0.167 |
| Random forest | 0.810 | 0.109 | 0.052 | 0.997 | 0.298 | 0.980 | 0.089 |  | 0.797 | 0.114 | 0.043 | 0.997 | 0.272 | 0.976 | 0.075 |  | 0.800 | 0.120 | 0.039 | 0.997 | 0.264 | 0.972 | 0.067 |
| Boost trees | 0.844 | 0.150 | 0.692 | 0.832 | 0.083 | 0.992 | 0.148 |  | 0.826 | 0.150 | 0.657 | 0.827 | 0.087 | 0.990 | 0.154 |  | 0.831 | 0.164 | 0.691 | 0.807 | 0.097 | 0.989 | 0.170 |
| MLP | 0.838 | 0.144 | 0.726 | 0.805 | 0.075 | 0.993 | 0.136 |  | 0.831 | 0.141 | 0.731 | 0.791 | 0.081 | 0.991 | 0.146 |  | 0.832 | 0.153 | 0.737 | 0.774 | 0.089 | 0.990 | 0.159 |
| MARS | 0.853 | 0.181 | 0.746 | 0.819 | 0.082 | 0.993 | 0.149 |  | 0.838 | 0.154 | 0.702 | 0.814 | 0.087 | 0.991 | 0.155 |  | 0.843 | 0.162 | 0.745 | 0.793 | 0.098 | 0.990 | 0.173 |
| KNN | 0.605 | 0.097 | 0.078 | 0.983 | 0.092 | 0.980 | 0.085 |  | 0.616 | 0.116 | 0.090 | 0.983 | 0.116 | 0.977 | 0.101 |  | 0.607 | 0.119 | 0.091 | 0.981 | 0.127 | 0.973 | 0.106 |
| BART | 0.794 | 0.109 | 0.543 | 0.868 | 0.082 | 0.989 | 0.143 |  | 0.784 | 0.113 | 0.524 | 0.861 | 0.087 | 0.986 | 0.149 |  | 0.788 | 0.122 | 0.550 | 0.850 | 0.099 | 0.984 | 0.168 |
|  | | | | | | | | | | | | | | | | | | | | | | | |
| **Predicting next ED visit >= 3** | | | | | | | | | | | | | | | | | | | | | | | |
| **Train-validation (2019-2020)** | | | | | | | |  | **Test (2020-2021)** | | | | | | |  | **Test (2021-2022)** | | | | | | |
|  | Model results (AUC) | PR AUC | Sensitivity | Specificity | Positive predictive value | Negative predictive value | F1 |  | Model results (AUC) | PR AUC | Sensitivity | Specificity | Positive predictive value | Negative predictive value | F1 |  | Model results (AUC) | PR AUC | Sensitivity | Specificity | Positive predictive value | Negative predictive value | F1 |
| Logistic regression | 0.838 | 0.272 | 0.718 | 0.822 | 0.139 | 0.986 | 0.233 |  | 0.831 | 0.245 | 0.698 | 0.805 | 0.128 | 0.985 | 0.216 |  | 0.820 | 0.260 | 0.696 | 0.789 | 0.146 | 0.980 | 0.241 |
| Random forest | 0.803 | 0.197 | 0.122 | 0.991 | 0.353 | 0.966 | 0.181 |  | 0.785 | 0.186 | 0.127 | 0.990 | 0.336 | 0.965 | 0.184 |  | 0.776 | 0.199 | 0.111 | 0.990 | 0.358 | 0.956 | 0.170 |
| Boost trees | 0.828 | 0.253 | 0.703 | 0.816 | 0.133 | 0.986 | 0.224 |  | 0.818 | 0.229 | 0.687 | 0.799 | 0.123 | 0.984 | 0.209 |  | 0.809 | 0.246 | 0.678 | 0.142 | 0.142 | 0.979 | 0.234 |
| MLP | 0.834 | 0.248 | 0.737 | 0.795 | 0.126 | 0.987 | 0.216 |  | 0.823 | 0.216 | 0.718 | 0.775 | 0.116 | 0.985 | 0.199 |  | 0.815 | 0.244 | 0.720 | 0.762 | 0.135 | 0.981 | 0.228 |
| MARS | 0.841 | 0.259 | 0.729 | 0.805 | 0.130 | 0.987 | 0.221 |  | 0.832 | 0.232 | 0.717 | 0.793 | 0.124 | 0.986 | 0.212 |  | 0.820 | 0.246 | 0.711 | 0.774 | 0.140 | 0.981 | 0.234 |
| KNN | 0.634 | 0.148 | 0.114 | 0.973 | 0.147 | 0.965 | 0.129 |  | 0.637 | 0.162 | 0.139 | 0.969 | 0.157 | 0.965 | 0.147 |  | 0.623 | 0.161 | 0.121 | 0.967 | 0.158 | 0.955 | 0.137 |
| BART | 0.799 | 0.224 | 0.621 | 0.843 | 0.137 | 0.982 | 0.225 |  | 0.784 | 0.185 | 0.613 | 0.824 | 0.125 | 0.981 | 0.207 |  | 0.775 | 0.203 | 0.598 | 0.818 | 0.145 | 0.975 | 0.234 |
|  | | | | | | | | | | | | | | | | | | | | | | | |
| **Predicting next ED visit >= 5** | | | | | | | | | | | | | | | | | | | | | | | |
| **Train-validation (2019-2020)** | | | | | | | |  | **Test (2020-2021)** | | | | | | |  | **Test (2021-2022)** | | | | | | |
|  | Model results (AUC) | PR AUC | Sensitivity | Specificity | Positive predictive value | Negative predictive value | F1 |  | Model results (AUC) | PR AUC | Sensitivity | Specificity | Positive predictive value | Negative predictive value | F1 |  | Model results (AUC) | PR AUC | Sensitivity | Specificity | Positive predictive value | Negative predictive value | F1 |
| Logistic regression | 0.892 | 0.210 | 0.780 | 0.864 | 0.067 | 0.997 | 0.123 |  | 0.892 | 0.195 | 0.767 | 0.857 | 0.058 | 0.997 | 0.108 |  | 0.877 | 0.209 | 0.762 | 0.837 | 0.066 | 0.996 | 0.121 |
| Random forest | 0.836 | 0.127 | 0.036 | 0.999 | 0.314 | 0.988 | 0.064 |  | 0.832 | 0.130 | 0.073 | 0.999 | 0.429 | 0.990 | 0.125 |  | 0.830 | 0.129 | 0.043 | 0.999 | 0.355 | 0.986 | 0.077 |
| Boost trees | 0.872 | 0.168 | 0.689 | 0.887 | 0.071 | 0.996 | 0.128 |  | 0.862 | 0.162 | 0.664 | 0.882 | 0.060 | 0.996 | 0.110 |  | 0.852 | 0.186 | 0.659 | 0.870 | 0.071 | 0.994 | 0.128 |
| MLP | 0.854 | 0.147 | 0.715 | 0.851 | 0.057 | 0.996 | 0.105 |  | 0.854 | 0.133 | 0.721 | 0.840 | 0.049 | 0.996 | 0.092 |  | 0.841 | 0.155 | 0.703 | 0.830 | 0.059 | 0.995 | 0.108 |
| MARS | 0.889 | 0.193 | 0.793 | 0.859 | 0.065 | 0.997 | 0.121 |  | 0.891 | 0.182 | 0.760 | 0.854 | 0.056 | 0.997 | 0.104 |  | 0.877 | 0.196 | 0.756 | 0.837 | 0.065 | 0.996 | 0.120 |
| KNN | 0.601 | 0.083 | 0.061 | 0.992 | 0.088 | 0.988 | 0.072 |  | 0.611 | 0.102 | 0.098 | 0.992 | 0.125 | 0.990 | 0.110 |  | 0.594 | 0.089 | 0.075 | 0.990 | 0.106 | 0.986 | 0.088 |
| BART | 0.800 | 0.102 | 0.463 | 0.927 | 0.073 | 0.993 | 0.127 |  | 0.795 | 0.104 | 0.484 | 0.923 | 0.067 | 0.994 | 0.118 |  | 0.787 | 0.120 | 0.467 | 0.915 | 0.077 | 0.991 | 0.132 |
|  | | | | | | | | | | | | | | | | | | | | | | | |
| **Predicting next ED visit >= 10** | | | | | | | | | | | | | | | | | | | | | | | |
| **Train-validation (2019-2020)** | | | | | | | |  | **Test (2020-2021)** | | | | | | |  | **Test (2021-2022)** | | | | | | |
|  | Model results (AUC) | PR AUC | Sensitivity | Specificity | Positive predictive value | Negative predictive value | F1 |  | Model results (AUC) | PR AUC | Sensitivity | Specificity | Positive predictive value | Negative predictive value | F1 |  | Model results (AUC) | PR AUC | Sensitivity | Specificity | Positive predictive value | Negative predictive value | F1 |
| Logistic regression | 0.914 | 0.225 | 0.714 | 0.940 | 0.020 | 0.999 | 0.038 |  | 0.936 | 0.236 | 0.759 | 0.936 | 0.018 | 1.000 | 0.035 |  | 0.938 | 0.186 | 0.800 | 0.934 | 0.020 | 1.000 | 0.039 |
| Random forest | 0.908 | 0.122 | 0.024 | 1.000 | 1.000 | 0.998 | 0.047 |  | 0.920 | 0.149 | 0.078 | 1.000 | 0.433 | 0.999 | 0.133 |  | 0.914 | 0.125 | 0.047 | 1.000 | 0.391 | 0.998 | 0.085 |
| Boost trees | 0.843 | 0.167 | 0.500 | 0.988 | 0.064 | 0.999 | 0.114 |  | 0.902 | 0.203 | 0.446 | 0.987 | 0.051 | 0.999 | 0.092 |  | 0.887 | 0.178 | 0.458 | 0.986 | 0.055 | 0.999 | 0.098 |
| MLP | 0.894 | 0.132 | 0.595 | 0.976 | 0.040 | 0.999 | 0.075 |  | 0.868 | 0.074 | 0.560 | 0.974 | 0.032 | 0.999 | 0.061 |  | 0.876 | 0.091 | 0.558 | 0.970 | 0.031 | 0.999 | 0.059 |
| MARS | 0.921 | 0.226 | 0.714 | 0.942 | 0.020 | 0.999 | 0.039 |  | 0.926 | 0.202 | 0.747 | 0.939 | 0.018 | 1.000 | 0.036 |  | 0.928 | 0.156 | 0.795 | 0.935 | 0.020 | 1.000 | 0.040 |
| KNN | 0.545 | 0.031 | 0.024 | 0.999 | 0.038 | 0.998 | 0.029 |  | 0.582 | 0.076 | 0.084 | 0.999 | 0.104 | 0.999 | 0.093 |  | 0.529 | 0.027 | 0.032 | 0.999 | 0.044 | 0.998 | 0.037 |
| BART | 0.864 | 0.153 | 0.405 | 0.992 | 0.075 | 0.999 | 0.127 |  | 0.848 | 0.168 | 0.416 | 0.991 | 0.068 | 0.999 | 0.117 |  | 0.848 | 0.101 | 0.384 | 0.991 | 0.067 | 0.999 | 0.114 |

**Table S6. Model performance in validation and test datasets for models trained with SMOTE (k = 3).**

| **Predicting next year stay >= 7 days** | | | | | | | | | | | | | | | | | | | | | | | |
| --- | --- | --- | --- | --- | --- | --- | --- | --- | --- | --- | --- | --- | --- | --- | --- | --- | --- | --- | --- | --- | --- | --- | --- |
| **Train-validation (2019-2020)** | | | | | | | |  | **Test (2020-2021)** | | | | | | |  | **Test (2021-2022)** | | | | | | |
|  | Model results (AUC) | PR AUC | Sensitivity | Specificity | Positive predictive value | Negative predictive value | F1 |  | Model results (AUC) | PR AUC | Sensitivity | Specificity | Positive predictive value | Negative predictive value | F1 |  | Model results (AUC) | PR AUC | Sensitivity | Specificity | Positive predictive value | Negative predictive value | F1 |
| Logistic regression | 0.777 | 0.294 | 0.651 | 0.769 | 0.200 | 0.961 | 0.306 |  | 0.778 | 0.290 | 0.675 | 0.752 | 0.209 | 0.960 | 0.319 |  | 0.772 | 0.307 | 0.674 | 0.744 | 0.229 | 0.953 | 0.342 |
| Random forest | 0.775 | 0.263 | 0.353 | 0.924 | 0.292 | 0.941 | 0.319 |  | 0.769 | 0.264 | 0.339 | 0.920 | 0.292 | 0.935 | 0.314 |  | 0.770 | 0.284 | 0.367 | 0.909 | 0.311 | 0.927 | 0.337 |
| Boost trees | 0.774 | 0.280 | 0.431 | 0.897 | 0.270 | 0.947 | 0.332 |  | 0.776 | 0.289 | 0.430 | 0.892 | 0.278 | 0.942 | 0.338 |  | 0.774 | 0.308 | 0.459 | 0.873 | 0.290 | 0.935 | 0.356 |
| MLP | 0.778 | 0.250 | 0.653 | 0.776 | 0.206 | 0.962 | 0.313 |  | 0.776 | 0.260 | 0.663 | 0.752 | 0.206 | 0.958 | 0.315 |  | 0.770 | 0.276 | 0.666 | 0.746 | 0.228 | 0.952 | 0.340 |
| MARS | 0.766 | 0.289 | 0.460 | 0.888 | 0.267 | 0.949 | 0.338 |  | 0.760 | 0.281 | 0.457 | 0.878 | 0.266 | 0.943 | 0.336 |  | 0.758 | 0.302 | 0.474 | 0.863 | 0.280 | 0.936 | 0.352 |
| KNN | 0.661 | 0.219 | 0.320 | 0.840 | 0.151 | 0.933 | 0.205 |  | 0.666 | 0.229 | 0.330 | 0.844 | 0.171 | 0.928 | 0.225 |  | 0.666 | 0.250 | 0.336 | 0.835 | 0.186 | 0.918 | 0.240 |
| BART | 0.746 | 0.237 | 0.542 | 0.808 | 0.201 | 0.952 | 0.293 |  | 0.742 | 0.237 | 0.544 | 0.791 | 0.202 | 0.947 | 0.294 |  | 0.741 | 0.257 | 0.559 | 0.781 | 0.223 | 0.940 | 0.319 |
|  | | | | | | | | | | | | | | | | | | | | | | | |
| **Predicting next year stay >= 14 days** | | | | | | | | | | | | | | | | | | | | | | | |
| **Train-validation (2019-2020)** | | | | | | | |  | **Test (2020-2021)** | | | | | | |  | **Test (2021-2022)** | | | | | | |
|  | Model results (AUC) | PR AUC | Sensitivity | Specificity | Positive predictive value | Negative predictive value | F1 |  | Model results (AUC) | PR AUC | Sensitivity | Specificity | Positive predictive value | Negative predictive value | F1 |  | Model results (AUC) | PR AUC | Sensitivity | Specificity | Positive predictive value | Negative predictive value | F1 |
| Logistic regression | 0.790 | 0.220 | 0.656 | 0.787 | 0.136 | 0.978 | 0.226 |  | 0.786 | 0.221 | 0.672 | 0.769 | 0.143 | 0.976 | 0.236 |  | 0.783 | 0.232 | 0.678 | 0.760 | 0.159 | 0.972 | 0.257 |
| Random forest | 0.787 | 0.184 | 0.282 | 0.947 | 0.216 | 0.963 | 0.245 |  | 0.776 | 0.183 | 0.259 | 0.949 | 0.227 | 0.957 | 0.242 |  | 0.779 | 0.198 | 0.287 | 0.938 | 0.235 | 0.952 | 0.258 |
| Boost trees | 0.792 | 0.214 | 0.458 | 0.911 | 0.208 | 0.970 | 0.287 |  | 0.790 | 0.223 | 0.431 | 0.909 | 0.214 | 0.965 | 0.286 |  | 0.789 | 0.237 | 0.460 | 0.892 | 0.221 | 0.961 | 0.299 |
| MLP | 0.789 | 0.202 | 0.651 | 0.794 | 0.139 | 0.978 | 0.230 |  | 0.782 | 0.199 | 0.646 | 0.779 | 0.143 | 0.975 | 0.235 |  | 0.777 | 0.212 | 0.654 | 0.764 | 0.156 | 0.971 | 0.252 |
| MARS | 0.771 | 0.206 | 0.470 | 0.898 | 0.192 | 0.971 | 0.272 |  | 0.759 | 0.203 | 0.433 | 0.893 | 0.188 | 0.965 | 0.263 |  | 0.763 | 0.220 | 0.459 | 0.878 | 0.202 | 0.960 | 0.280 |
| KNN | 0.667 | 0.167 | 0.268 | 0.890 | 0.111 | 0.959 | 0.157 |  | 0.673 | 0.179 | 0.277 | 0.891 | 0.127 | 0.956 | 0.174 |  | 0.667 | 0.188 | 0.275 | 0.884 | 0.136 | 0.948 | 0.182 |
| BART | 0.738 | 0.163 | 0.492 | 0.838 | 0.135 | 0.970 | 0.212 |  | 0.731 | 0.160 | 0.482 | 0.827 | 0.138 | 0.965 | 0.214 |  | 0.734 | 0.175 | 0.509 | 0.813 | 0.154 | 0.961 | 0.237 |
|  | | | | | | | | | | | | | | | | | | | | | | | |
| **Predicting next year stay >= 14 days** | | | | | | | | | | | | | | | | | | | | | | | |
| **Train-validation (2019-2020)** | | | | | | | |  | **Test (2020-2021)** | | | | | | |  | **Test (2021-2022)** | | | | | | |
|  | Model results (AUC) | PR AUC | Sensitivity | Specificity | Positive predictive value | Negative predictive value | F1 |  | Model results (AUC) | PR AUC | Sensitivity | Specificity | Positive predictive value | Negative predictive value | F1 |  | Model results (AUC) | PR AUC | Sensitivity | Specificity | Positive predictive value | Negative predictive value | F1 |
| Logistic regression | 0.804 | 0.149 | 0.657 | 0.808 | 0.069 | 0.991 | 0.125 |  | 0.786 | 0.134 | 0.638 | 0.794 | 0.072 | 0.989 | 0.130 |  | 0.791 | 0.139 | 0.675 | 0.776 | 0.083 | 0.988 | 0.148 |
| Random forest | 0.800 | 0.100 | 0.159 | 0.977 | 0.131 | 0.982 | 0.143 |  | 0.777 | 0.089 | 0.120 | 0.981 | 0.138 | 0.978 | 0.128 |  | 0.780 | 0.101 | 0.152 | 0.974 | 0.148 | 0.975 | 0.150 |
| Boost trees | 0.805 | 0.131 | 0.442 | 0.927 | 0.116 | 0.987 | 0.184 |  | 0.791 | 0.128 | 0.406 | 0.930 | 0.127 | 0.984 | 0.193 |  | 0.795 | 0.138 | 0.436 | 0.909 | 0.126 | 0.982 | 0.196 |
| MLP | 0.798 | 0.111 | 0.659 | 0.818 | 0.073 | 0.991 | 0.132 |  | 0.773 | 0.108 | 0.621 | 0.806 | 0.075 | 0.988 | 0.134 |  | 0.782 | 0.120 | 0.663 | 0.782 | 0.084 | 0.987 | 0.148 |
| MARS | 0.788 | 0.140 | 0.463 | 0.909 | 0.100 | 0.987 | 0.164 |  | 0.770 | 0.125 | 0.453 | 0.904 | 0.107 | 0.985 | 0.173 |  | 0.771 | 0.132 | 0.463 | 0.889 | 0.111 | 0.982 | 0.179 |
| KNN | 0.652 | 0.118 | 0.181 | 0.945 | 0.067 | 0.981 | 0.097 |  | 0.652 | 0.116 | 0.178 | 0.945 | 0.076 | 0.979 | 0.107 |  | 0.651 | 0.122 | 0.178 | 0.938 | 0.080 | 0.974 | 0.110 |
| BART | 0.719 | 0.081 | 0.379 | 0.887 | 0.068 | 0.985 | 0.116 |  | 0.701 | 0.076 | 0.363 | 0.884 | 0.073 | 0.982 | 0.122 |  | 0.708 | 0.089 | 0.399 | 0.864 | 0.081 | 0.980 | 0.135 |
|  | | | | | | | | | | | | | | | | | | | | | | | |
| **Predicting next ED visit >= 3** | | | | | | | | | | | | | | | | | | | | | | | |
| **Train-validation (2019-2020)** | | | | | | | |  | **Test (2020-2021)** | | | | | | |  | **Test (2021-2022)** | | | | | | |
|  | Model results (AUC) | PR AUC | Sensitivity | Specificity | Positive predictive value | Negative predictive value | F1 |  | Model results (AUC) | PR AUC | Sensitivity | Specificity | Positive predictive value | Negative predictive value | F1 |  | Model results (AUC) | PR AUC | Sensitivity | Specificity | Positive predictive value | Negative predictive value | F1 |
| Logistic regression | 0.800 | 0.244 | 0.648 | 0.830 | 0.133 | 0.983 | 0.221 |  | 0.782 | 0.216 | 0.633 | 0.812 | 0.122 | 0.982 | 0.204 |  | 0.771 | 0.237 | 0.627 | 0.801 | 0.140 | 0.976 | 0.229 |
| Random forest | 0.793 | 0.184 | 0.284 | 0.964 | 0.238 | 0.971 | 0.259 |  | 0.771 | 0.166 | 0.248 | 0.965 | 0.225 | 0.969 | 0.236 |  | 0.759 | 0.179 | 0.257 | 0.957 | 0.234 | 0.961 | 0.245 |
| Boost trees | 0.806 | 0.235 | 0.460 | 0.935 | 0.222 | 0.977 | 0.299 |  | 0.791 | 0.205 | 0.413 | 0.937 | 0.211 | 0.975 | 0.279 |  | 0.780 | 0.228 | 0.415 | 0.924 | 0.221 | 0.968 | 0.289 |
| MLP | 0.783 | 0.216 | 0.638 | 0.806 | 0.117 | 0.982 | 0.197 |  | 0.765 | 0.186 | 0.630 | 0.794 | 0.111 | 0.981 | 0.189 |  | 0.754 | 0.206 | 0.623 | 0.774 | 0.125 | 0.975 | 0.208 |
| MARS | 0.787 | 0.235 | 0.455 | 0.930 | 0.207 | 0.977 | 0.285 |  | 0.767 | 0.199 | 0.419 | 0.925 | 0.186 | 0.975 | 0.257 |  | 0.755 | 0.221 | 0.410 | 0.920 | 0.209 | 0.968 | 0.277 |
| KNN | 0.663 | 0.151 | 0.254 | 0.907 | 0.099 | 0.968 | 0.143 |  | 0.658 | 0.147 | 0.249 | 0.906 | 0.098 | 0.967 | 0.141 |  | 0.639 | 0.152 | 0.225 | 0.897 | 0.101 | 0.957 | 0.140 |
| BART | 0.715 | 0.155 | 0.463 | 0.865 | 0.121 | 0.976 | 0.192 |  | 0.699 | 0.136 | 0.435 | 0.858 | 0.112 | 0.974 | 0.177 |  | 0.690 | 0.154 | 0.439 | 0.843 | 0.126 | 0.967 | 0.196 |
|  | | | | | | | | | | | | | | | | | | | | | | | |
| **Predicting next ED visit >= 5** | | | | | | | | | | | | | | | | | | | | | | | |
| **Train-validation (2019-2020)** | | | | | | | |  | **Test (2020-2021)** | | | | | | |  | **Test (2021-2022)** | | | | | | |
|  | Model results (AUC) | PR AUC | Sensitivity | Specificity | Positive predictive value | Negative predictive value | F1 |  | Model results (AUC) | PR AUC | Sensitivity | Specificity | Positive predictive value | Negative predictive value | F1 |  | Model results (AUC) | PR AUC | Sensitivity | Specificity | Positive predictive value | Negative predictive value | F1 |
| Logistic regression | 0.855 | 0.192 | 0.686 | 0.871 | 0.062 | 0.996 | 0.114 |  | 0.844 | 0.188 | 0.693 | 0.869 | 0.057 | 0.996 | 0.105 |  | 0.842 | 0.201 | 0.694 | 0.850 | 0.065 | 0.995 | 0.119 |
| Random forest | 0.831 | 0.095 | 0.152 | 0.989 | 0.150 | 0.989 | 0.151 |  | 0.822 | 0.094 | 0.142 | 0.992 | 0.164 | 0.990 | 0.152 |  | 0.820 | 0.103 | 0.159 | 0.989 | 0.179 | 0.987 | 0.169 |
| Boost trees | 0.842 | 0.163 | 0.417 | 0.955 | 0.104 | 0.992 | 0.166 |  | 0.837 | 0.146 | 0.440 | 0.958 | 0.107 | 0.993 | 0.172 |  | 0.833 | 0.156 | 0.455 | 0.948 | 0.116 | 0.991 | 0.185 |
| MLP | 0.780 | 0.108 | 0.602 | 0.860 | 0.051 | 0.994 | 0.093 |  | 0.798 | 0.123 | 0.632 | 0.854 | 0.047 | 0.995 | 0.088 |  | 0.788 | 0.119 | 0.621 | 0.842 | 0.056 | 0.993 | 0.102 |
| MARS | 0.843 | 0.164 | 0.515 | 0.951 | 0.115 | 0.994 | 0.188 |  | 0.822 | 0.162 | 0.513 | 0.949 | 0.104 | 0.994 | 0.173 |  | 0.824 | 0.178 | 0.506 | 0.946 | 0.124 | 0.992 | 0.200 |
| KNN | 0.663 | 0.093 | 0.149 | 0.970 | 0.058 | 0.989 | 0.084 |  | 0.660 | 0.099 | 0.173 | 0.972 | 0.066 | 0.990 | 0.096 |  | 0.647 | 0.092 | 0.143 | 0.967 | 0.062 | 0.987 | 0.087 |
| BART | 0.722 | 0.083 | 0.385 | 0.936 | 0.069 | 0.992 | 0.118 |  | 0.706 | 0.077 | 0.350 | 0.934 | 0.057 | 0.992 | 0.098 |  | 0.716 | 0.085 | 0.368 | 0.923 | 0.067 | 0.990 | 0.114 |
|  | | | | | | | | | | | | | | | | | | | | | | | |
| **Predicting next ED visit >= 10** | | | | | | | | | | | | | | | | | | | | | | | |
| **Train-validation (2019-2020)** | | | | | | | |  | **Test (2020-2021)** | | | | | | |  | **Test (2021-2022)** | | | | | | |
|  | Model results (AUC) | PR AUC | Sensitivity | Specificity | Positive predictive value | Negative predictive value | F1 |  | Model results (AUC) | PR AUC | Sensitivity | Specificity | Positive predictive value | Negative predictive value | F1 |  | Model results (AUC) | PR AUC | Sensitivity | Specificity | Positive predictive value | Negative predictive value | F1 |
| Logistic regression | 0.869 | 0.236 | 0.714 | 0.947 | 0.022 | 0.999 | 0.043 |  | 0.845 | 0.204 | 0.633 | 0.947 | 0.018 | 0.999 | 0.035 |  | 0.869 | 0.180 | 0.679 | 0.936 | 0.018 | 0.999 | 0.035 |
| Random forest | 0.914 | 0.047 | 0.048 | 0.999 | 0.133 | 0.998 | 0.070 |  | 0.911 | 0.079 | 0.102 | 0.999 | 0.227 | 0.999 | 0.141 |  | 0.897 | 0.064 | 0.074 | 0.999 | 0.126 | 0.998 | 0.093 |
| Boost trees | 0.915 | 0.192 | 0.429 | 0.991 | 0.075 | 0.999 | 0.127 |  | 0.917 | 0.202 | 0.410 | 0.992 | 0.073 | 0.999 | 0.123 |  | 0.910 | 0.138 | 0.400 | 0.990 | 0.065 | 0.999 | 0.112 |
| MLP | 0.875 | 0.195 | 0.524 | 0.970 | 0.028 | 0.999 | 0.053 |  | 0.835 | 0.086 | 0.416 | 0.966 | 0.019 | 0.999 | 0.035 |  | 0.846 | 0.073 | 0.458 | 0.963 | 0.021 | 0.999 | 0.040 |
| MARS | 0.897 | 0.239 | 0.619 | 0.965 | 0.029 | 0.999 | 0.055 |  | 0.857 | 0.139 | 0.596 | 0.964 | 0.025 | 0.999 | 0.048 |  | 0.866 | 0.155 | 0.658 | 0.961 | 0.028 | 0.999 | 0.054 |
| KNN | 0.578 | 0.039 | 0.071 | 0.997 | 0.035 | 0.998 | 0.047 |  | 0.612 | 0.074 | 0.139 | 0.997 | 0.070 | 0.999 | 0.093 |  | 0.585 | 0.048 | 0.074 | 0.996 | 0.030 | 0.998 | 0.043 |
| BART | 0.854 | 0.047 | 0.333 | 0.991 | 0.059 | 0.999 | 0.100 |  | 0.804 | 0.068 | 0.319 | 0.991 | 0.053 | 0.999 | 0.092 |  | 0.825 | 0.049 | 0.253 | 0.989 | 0.038 | 0.999 | 0.066 |

**Table S7. Top 10 variable importance score for selected models by outcomes.**

| **Predicting ≥ 7 inpatient bed days in subsequent year** | | | | | | |
| --- | --- | --- | --- | --- | --- | --- |
|  | Model | | | | | |
|  | Logistic regression | | Boost tree | | MARS | |
| Variable rank | Variable | Importance score | Variable | Importance score | Variable | Importance score |
| 1 | Age | 57.30 | ED visits (numerical) | 0.28 | ED visits (numerical) | 17 |
| 2 | ED visits (numerical) | 37.74 | Inpatient bed days (categorical) | 0.22 | Age | 16 |
| 3 | CKD Stage 5 | 29.50 | CKD Stage (categorical) | 0.14 | Nephropathy (present) | 15 |
| 4 | 7-13 inpatient bed days | 25.80 | Age | 0.14 | DM medication category (Oral and Insulin) | 14 |
| 5 | 14-29 inpatient bed days | 25.39 | Mean HbA1c (%) | 0.05 | CKD Stage 5 | 13 |
| 6 | CKD Stage 4 | 25.23 | Singapore Housing Index | 0.04 | IHD (present) | 12 |
| 7 | 3-6 inpatient bed days | 24.49 | DM medication category | 0.02 | Peripheral arterial disease (present) | 11 |
| 8 | IHD (present) | 20.50 | Nephropathy | 0.02 | CKD Stage 4 | 10 |
| 9 | Ischemic stroke (present) | 18.79 | IHD | 0.02 | Ischemic stroke (present) | 9 |
| 10 | Nephropathy (present) | 16.96 | Peripheral arterial disease | 0.02 | Singapore Housing Index | 8 |
|  | | | | | | |
| **Predicting ≥ 14 inpatient bed days in subsequent year** | | | | | | |
|  | Model | | | | | |
|  | Logistic regression | | Boost tree | | MARS | |
| Variable rank | Variable | Importance score | Variable | Importance score | Variable | Importance score |
| 1 | Age | 58.46 | ED visits (numerical) | 0.32 | ED visits (numerical) | 18 |
| 2 | ED visits (numerical) | 41.07 | Inpatient bed days (categorical) | 0.19 | Age | 17 |
| 3 | CKD Stage 5 | 30.41 | CKD Stage (categorical) | 0.14 | Nephropathy (present) | 16 |
| 4 | CKD Stage 4 | 28.57 | Age | 0.13 | DM medication category (Oral and Insulin) | 15 |
| 5 | 14-29 inpatient bed days | 25.94 | Mean HbA1c (%) | 0.06 | CKD Stage 5 | 14 |
| 6 | 7-13 inpatient bed days | 22.60 | Singapore Housing Index | 0.04 | IHD (present) | 13 |
| 7 | 3-6 inpatient bed days | 22.56 | DM medication category | 0.02 | CKD Stage 4 | 12 |
| 8 | ≥ 30 inpatient bed days | 22.04 | Peripheral arterial disease | 0.02 | Peripheral arterial disease (present) | 11 |
| 9 | IHD (present) | 18.97 | IHD | 0.02 | Mean HbA1c (%) | 10 |
| 10 | Peripheral arterial disease (present) | 18.91 | Nephropathy | 0.01 | Ischemic stroke (present) | 8 |
|  | | | | | | |
| **Predicting ≥ 30 inpatient bed days in subsequent year** | | | | | | |
|  | Model | | | | | |
|  | Logistic regression | | Boost tree | | MARS | |
| Variable rank | Variable | Importance score | Variable | Importance score | Variable | Importance score |
| 1 | Age | 55.88 | ED visits (numerical) | 0.40 | ED visits (numerical) | 18 |
| 2 | ED visits (numerical) | 45.54 | CKD Stage (categorical) | 0.12 | Nephropathy (present) | 17 |
| 3 | CKD Stage 5 | 29.75 | Age | 0.11 | Age | 16 |
| 4 | CKD Stage 4 | 28.63 | Inpatient bed days (categorical) | 0.09 | Peripheral arterial disease (present) | 14 |
| 5 | ≥ 30 inpatient bed days | 26.68 | Mean HbA1c (%) | 0.09 | DM medication category (Insulin only) | 14 |
| 6 | 14-29 inpatient bed days | 26.56 | Singapore Housing Index | 0.06 | DM medication category (Oral and Insulin) | 14 |
| 7 | Peripheral arterial disease (present) | 26.50 | DM medication category | 0.03 | Ischemic stroke (present) | 12 |
| 8 | Gender (Female) | 24.25 | Peripheral arterial disease | 0.02 | CKD Stage 5 | 11 |
| 9 | Ischemic stroke (present) | 23.66 | Ischemic stroke | 0.01 | Mean HbA1c (%) | 10 |
| 10 | Mean HbA1c (%) | 20.32 | Nephropathy | 0.01 | CKD Stage 4 | 8 |
|  | | | | | | |
| **Predicting ≥ 3 emergency department visits in subsequent year** | | | | | | |
|  | Model | | | | | |
|  | Logistic regression | | Boost tree | | MARS | |
| Variable rank | Variable | Importance score | Variable | Importance score | Variable | Importance score |
| 1 | ED visits (numerical) | 80.53 | ED visits (numerical) | 0.60 | ED visits (numerical) | 19 |
| 2 | CKD Stage 4 | 27.46 | CKD Stage (categorical) | 0.09 | IHD (present) | 17 |
| 3 | CKD Stage 5 | 26.88 | Mean HbA1c (%) | 0.07 | Nephropathy (present) | 16 |
| 4 | Age | 25.72 | Age | 0.06 | Mean HbA1c (%) | 16 |
| 5 | Mean HbA1c (%) | 22.52 | Singapore Housing Index | 0.06 | Age | 15 |
| 6 | IHD (present) | 21.58 | Inpatient bed days (categorical) | 0.02 | CKD Stage 4 | 14 |
| 7 | 3-6 inpatient bed days | 20.89 | DM medication category | 0.02 | CKD Stage 5 | 14 |
| 8 | Ethnicity (Indian) | 20.08 | Ethnicity | 0.02 | Singapore Housing Index | 13 |
| 9 | Ethnicity (Malay) | 19.66 | IHD | 0.02 | Ethnicity (Malay) | 7 |
| 10 | 7-13 inpatient bed days | 17.13 | Peripheral arterial disease | 0.01 | Ethnicity (Indian) | 7 |
|  | | | | | | |
| **Predicting ≥ 5 emergency department visits in subsequent year** | | | | | | |
|  | Model | | | | | |
|  | Logistic regression | | Boost tree | | MARS | |
| Variable rank | Variable | Importance score | Variable | Importance score | Variable | Importance score |
| 1 | ED visits (numerical) | 101.09 | ED visits (numerical) | 0.59 | ED visits (numerical) | 15 |
| 2 | Age | 38.12 | Mean HbA1c (%) | 0.09 | Mean HbA1c (%) | 14 |
| 3 | CKD Stage 5 | 29.76 | Age | 0.08 | Age | 13 |
| 4 | CKD Stage 4 | 27.55 | CKD Stage (categorical) | 0.07 | CKD Stage 5 | 12 |
| 5 | Mean HbA1c (%) | 24.52 | Singapore Housing Index | 0.06 | CKD Stage 4 | 11 |
| 6 | Ethnicity (Other) | 19.34 | DM medication category | 0.02 | Singapore Housing Index | 10 |
| 7 | Ethnicity (Indian) | 18.60 | Ethnicity | 0.01 | IHD (present) | 9 |
| 8 | Ethnicity (Malay) | 17.99 | IHD | 0.01 | CKD Stage 3A | 8 |
| 9 | Haemorrhagic stroke (present) | 15.64 | Ischemic stroke | 0.01 | DM medication category (Oral and Insulin) | 7 |
| 10 | Neuropathy (present) | 14.78 | Peripheral arterial disease | 0.01 | DM medication category (Insulin only) | 5 |
|  | | | | | | |
| **Predicting ≥ 10 emergency department visits in subsequent year** | | | | | | |
|  | Model | | | | | |
|  | Logistic regression | | Boost tree | | MARS | |
| Variable rank | Variable | Importance score | Variable | Importance score | Variable | Importance score |
| 1 | ED visits (numerical) | 105.41 | ED visits (numerical) | 0.70 | ED visits (numerical) | 29 |
| 2 | Peripheral arterial disease (present) | 47.60 | Mean HbA1c (%) | 0.08 | Peripheral arterial disease (present) | 26 |
| 3 | DM medication category (Oral only) | 47.46 | Age | 0.07 | Hyperlipidemia (present) | 25 |
| 4 | Hyperlipidemia (present) | 42.90 | Singapore Housing Index | 0.06 | Singapore Housing Index | 24 |
| 5 | CKD Stage 5 | 34.81 | Peripheral arterial disease | 0.03 | DM medication category (Oral only) | 23 |
| 6 | DM medication category (Oral and Insulin) | 31.87 | IHD | 0.01 | Age | 19 |
| 7 | Ethnicity (Other) | 28.50 | Inpatient bed days (categorical) | 0.01 | Diabetic eye disease (present) | 17 |
| 8 | DM medication category (Insulin only) | 25.78 | Ethnicity | 0.01 | Dialysis (present) | 15 |
| 9 | Rental block (present) | 25.38 | CKD Stage (categorical) | 0.01 | CKD Stage 5 | 13 |
| 10 | 1-2 inpatient bed days | 24.8 | Hyperlipidemia | 0.01 | Gender (Female) | 12 |

**Figure S1. Performance of models trained using SMOTE-NC (K=3) to predict inpatient bed days.**


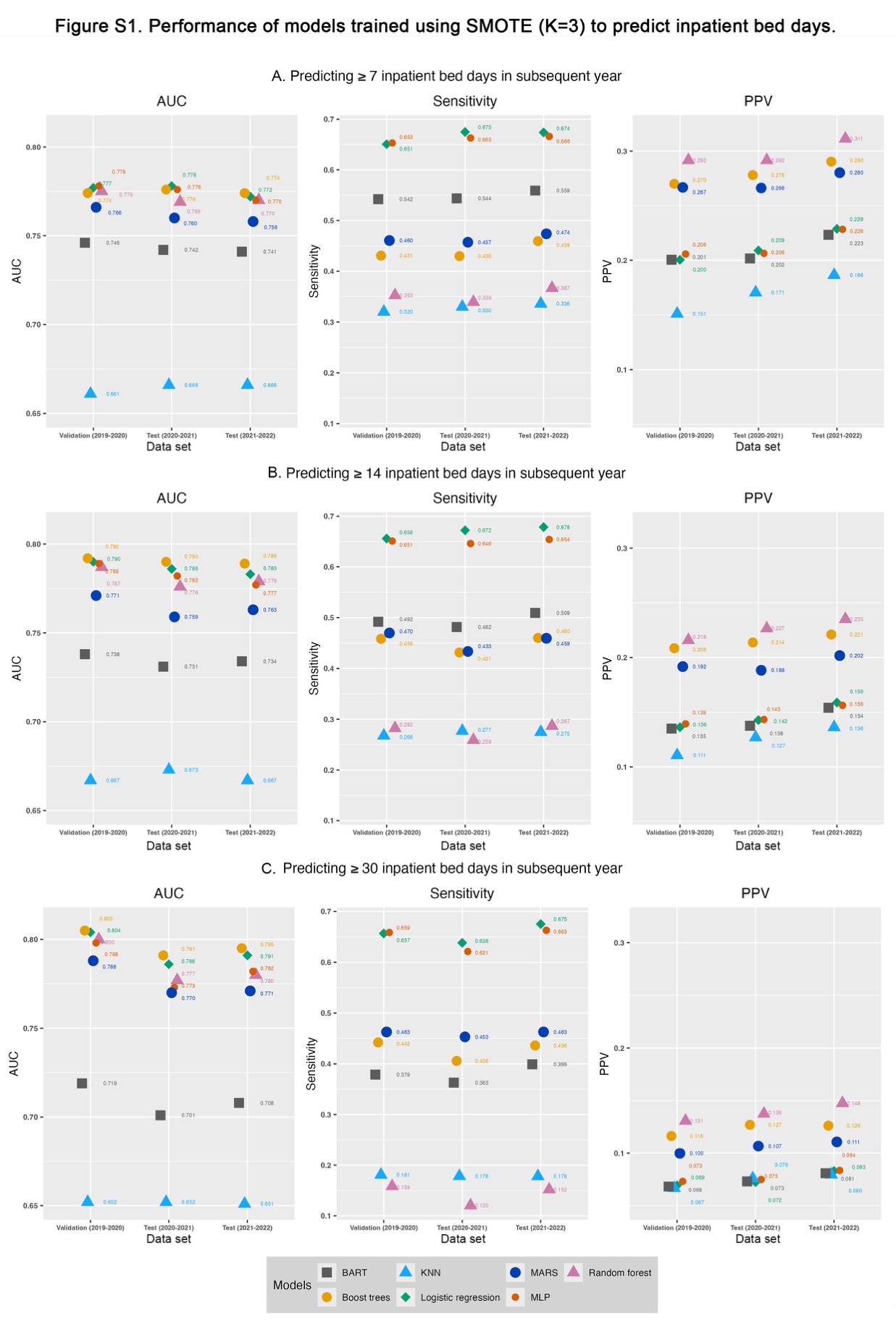


**Figure S2. Performance of models trained using SMOTE-NC (k=3) to predict emergency department visits.**


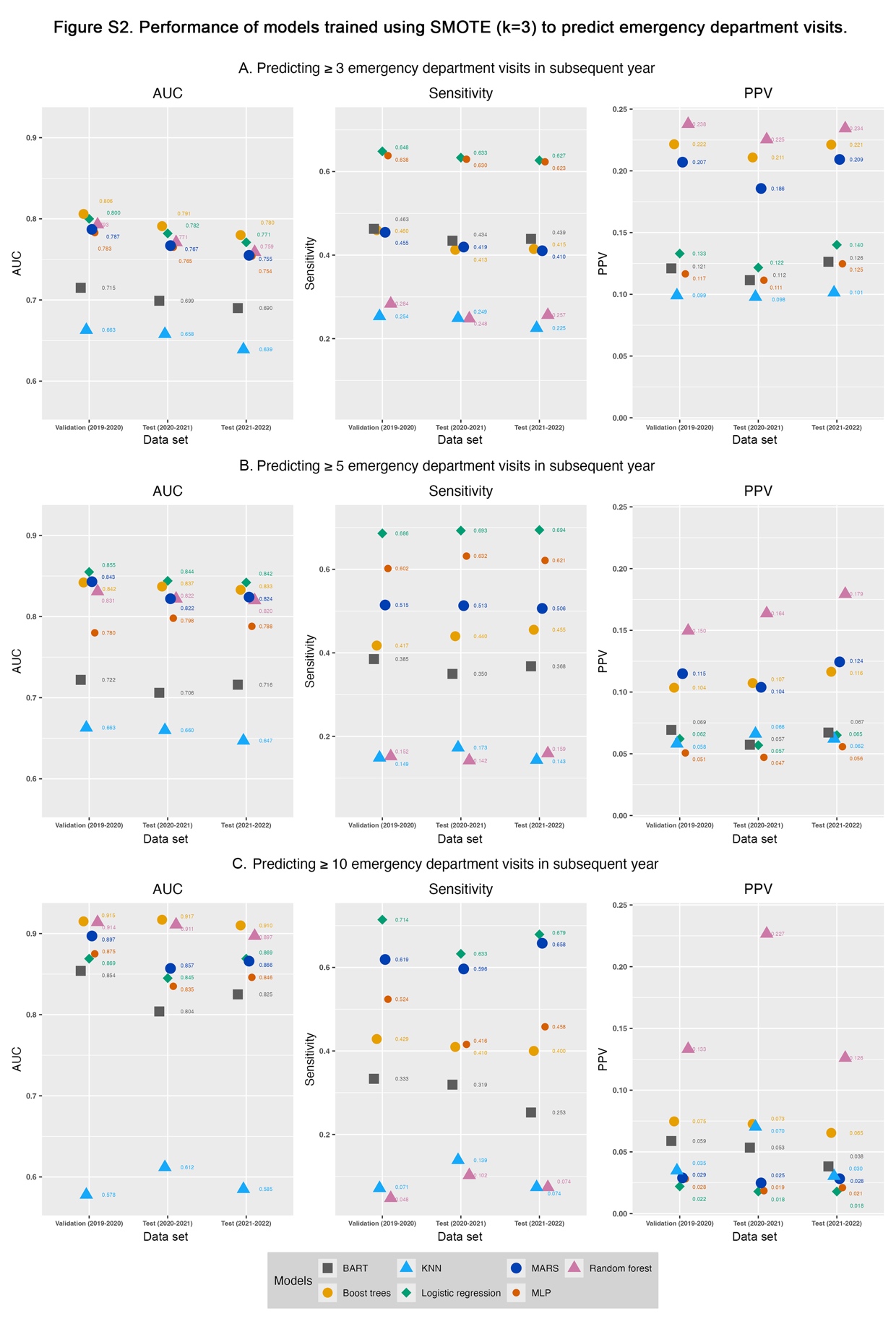


**Figure S3. Permutation feature importance plots for models trained using random over-sampling to predict inpatient bed days.**


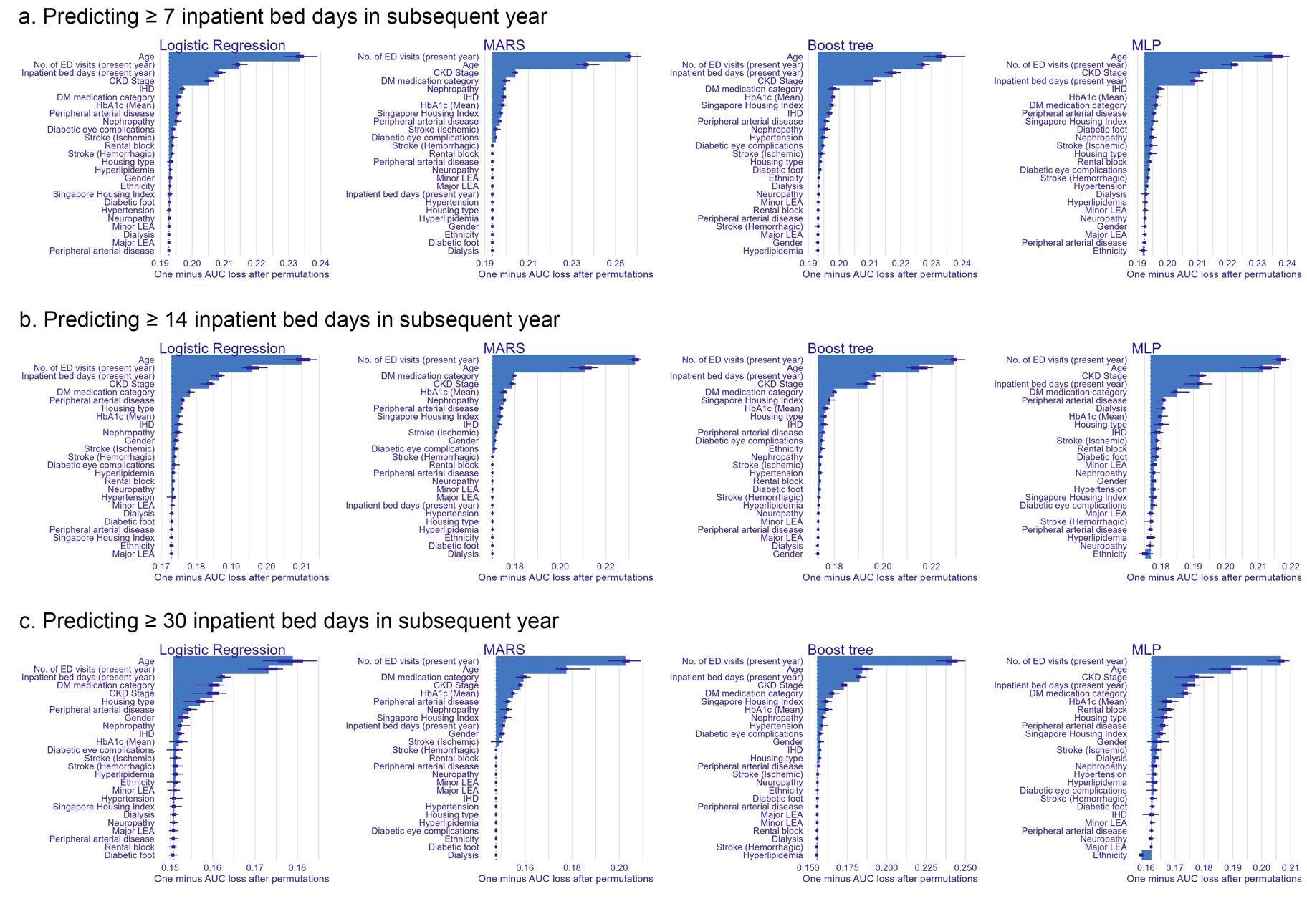


**Figure S4. Permutation feature importance plots for models trained using random over-sampling to predict emergency department visits.**

**
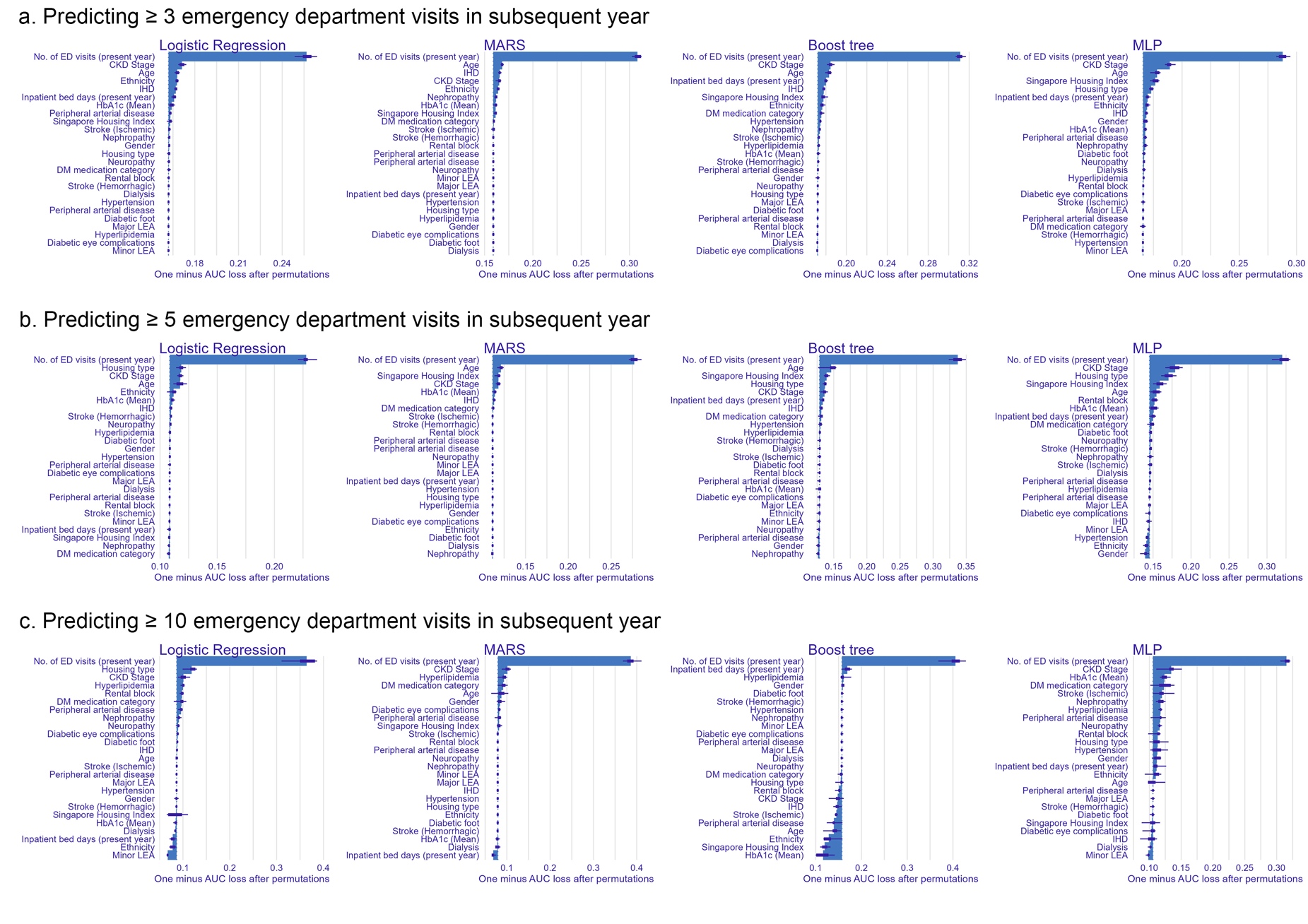
**

**Figure S5. Partial dependence plots for selected variables for models trained using random over-sampling to predict inpatient bed days ≥ 7 days.**


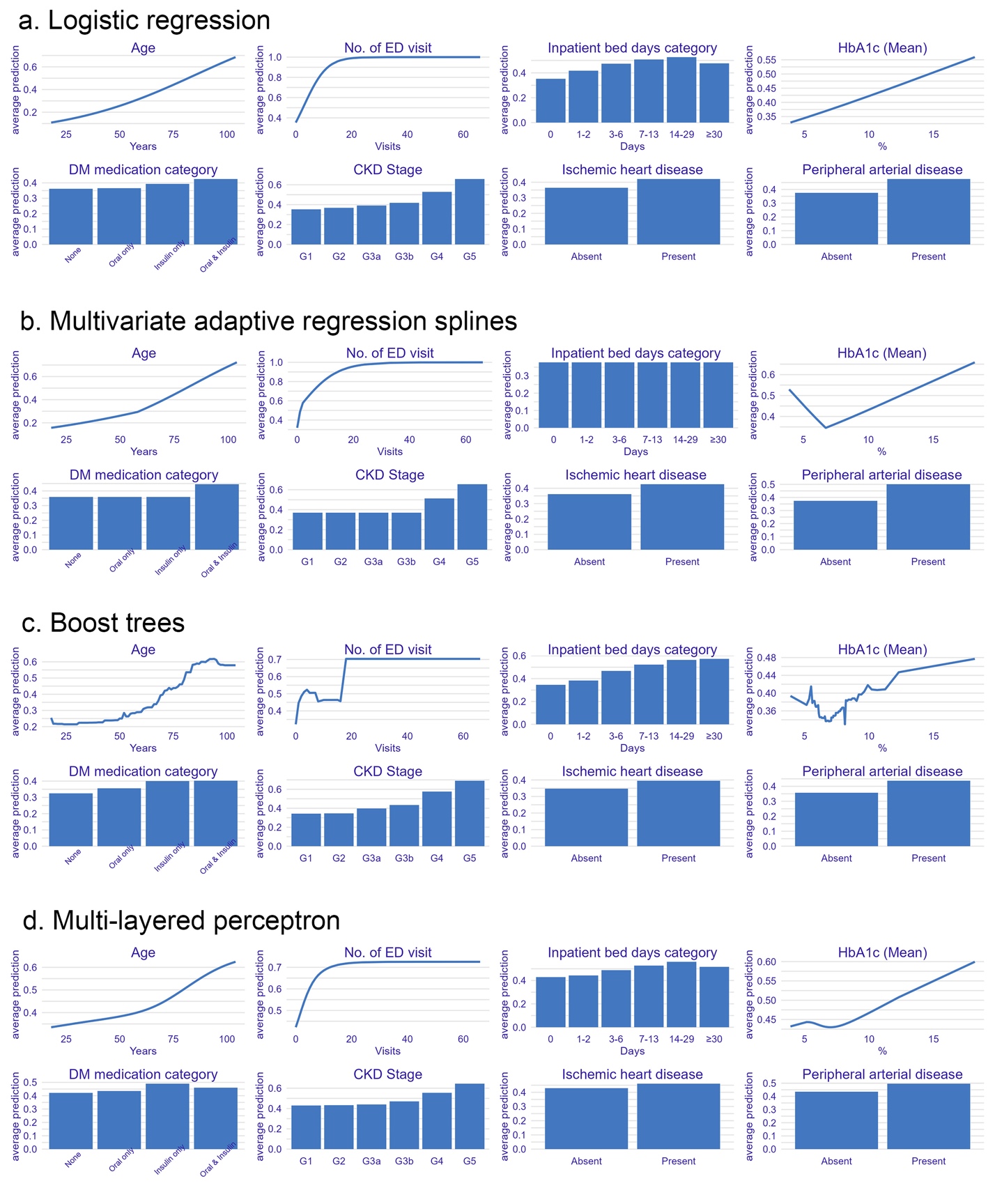


**Figure S6. Partial dependence plots for selected variables for models trained using random over-sampling to predict inpatient bed days ≥ 14 days.**


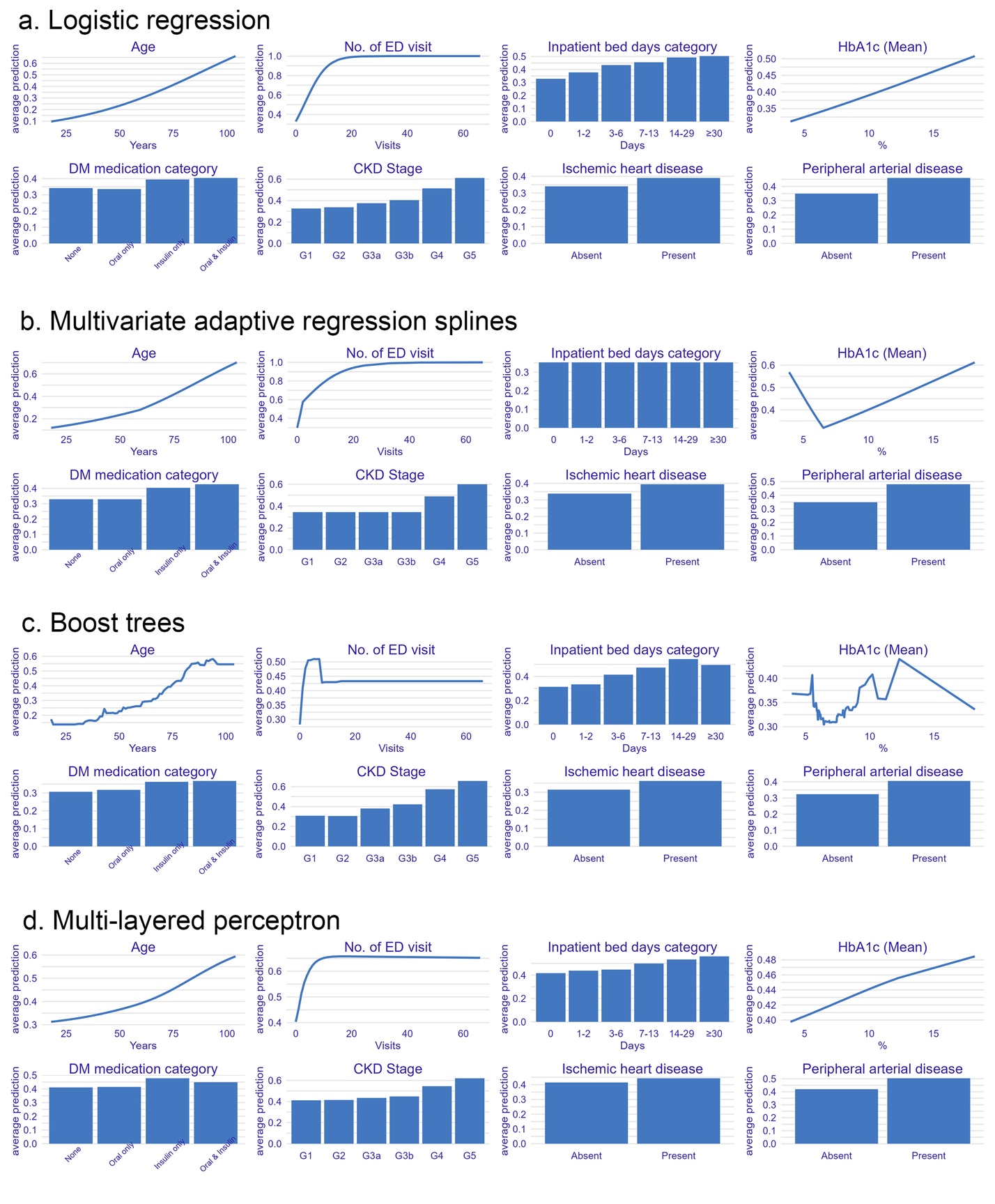


**Figure S7. Partial dependence plots for selected variables for models trained using random over-sampling to predict inpatient bed days ≥ 30 days.**


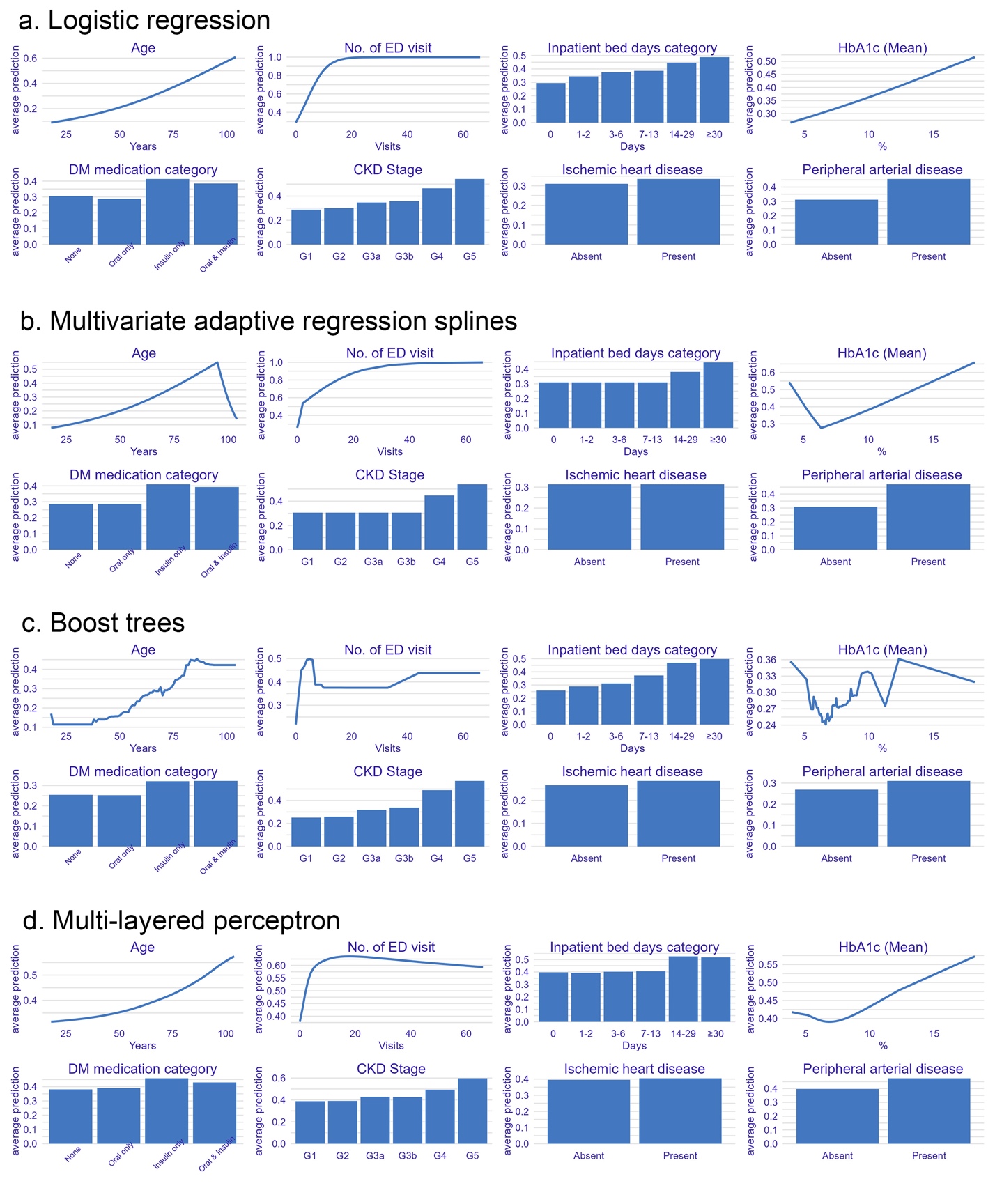


**Figure S8. Partial dependence plots for selected variables for models trained using random over-sampling to predict emergency department visits ≥ 3 visits.**


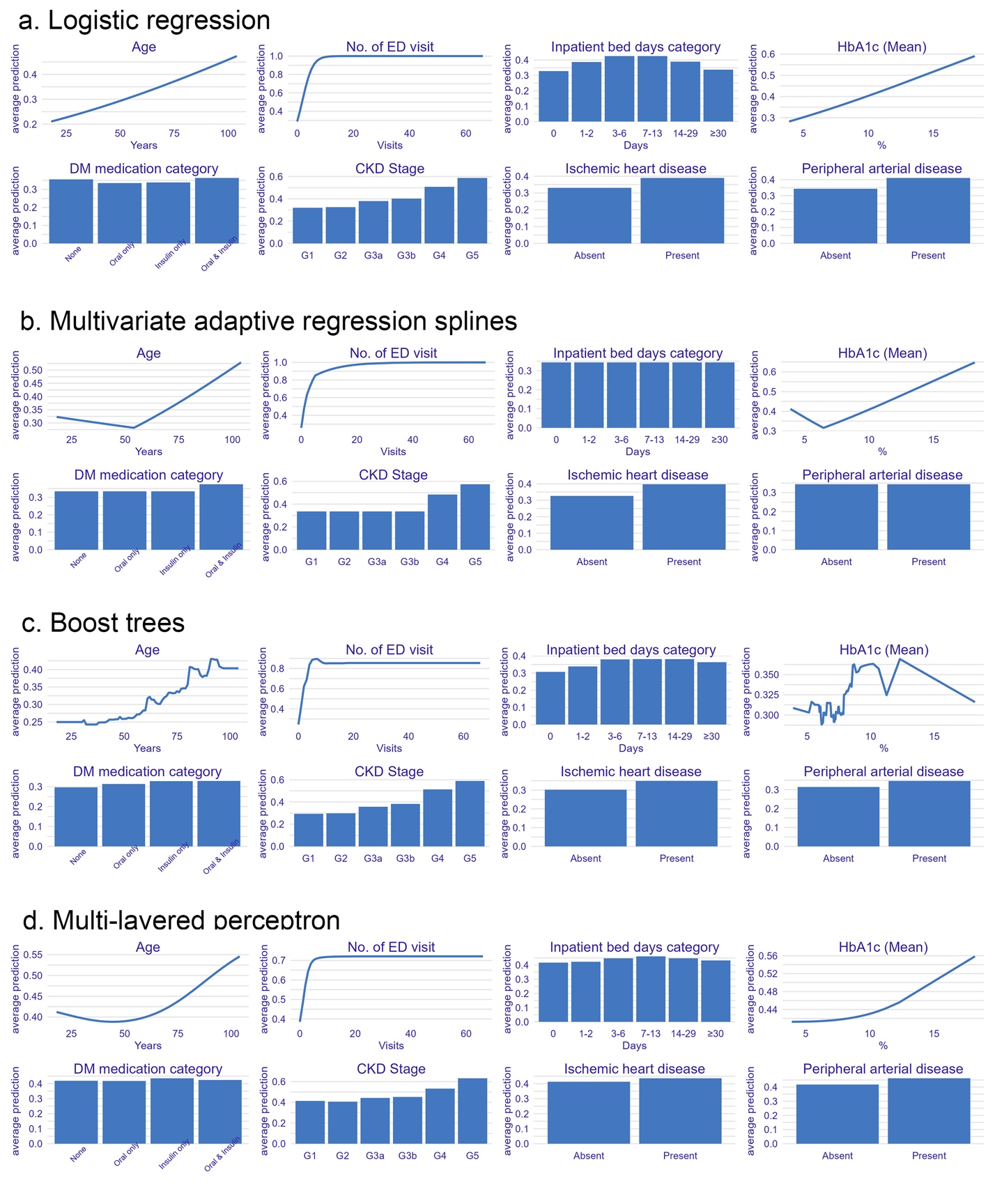


**Figure S9. Partial dependence plots for selected variables for models trained using random over-sampling to predict emergency department visits ≥ 5 visits.**


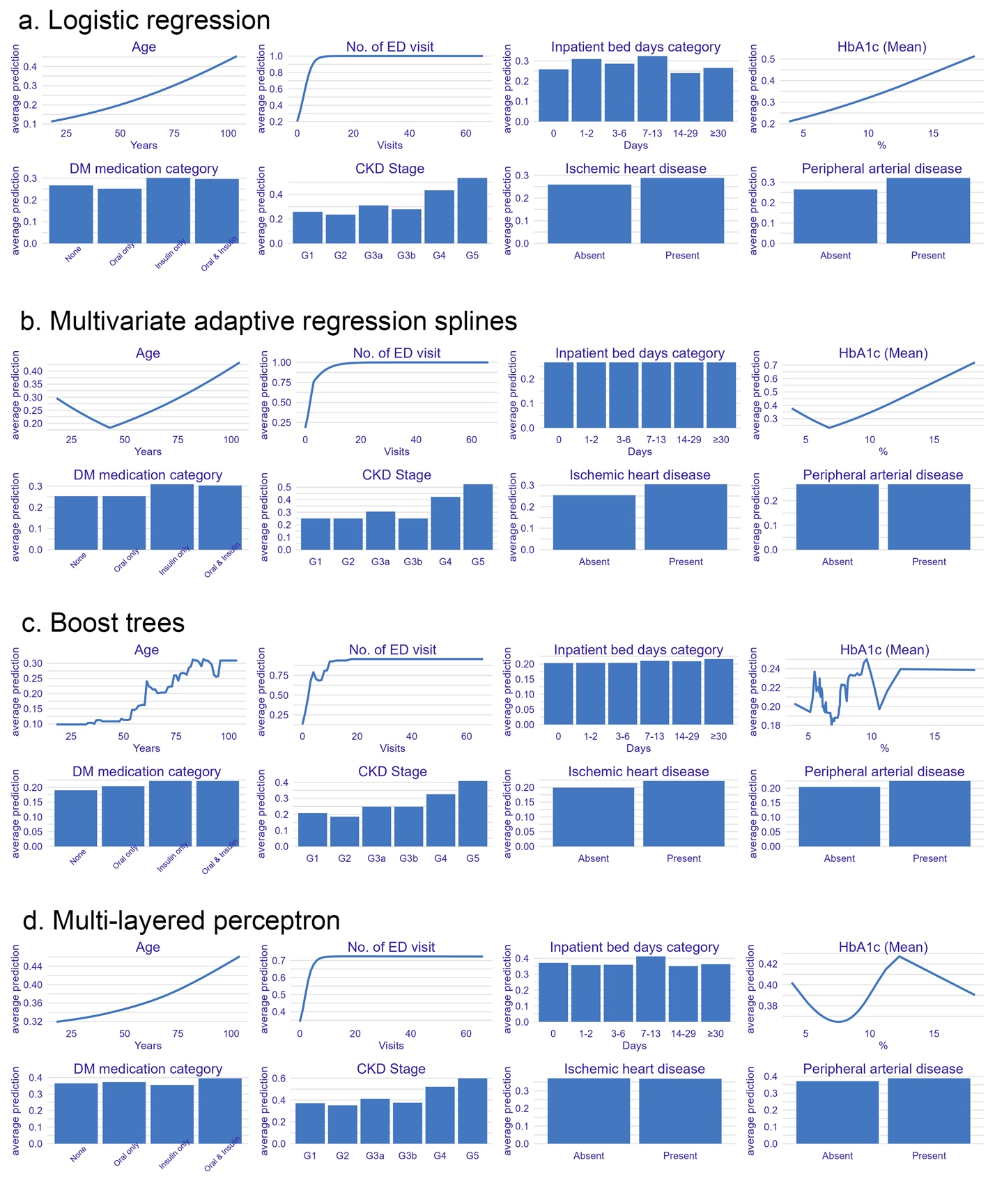


**Figure S10. Partial dependence plots for selected variables for models trained using random over-sampling to predict emergency department visits ≥ 10 visits.**


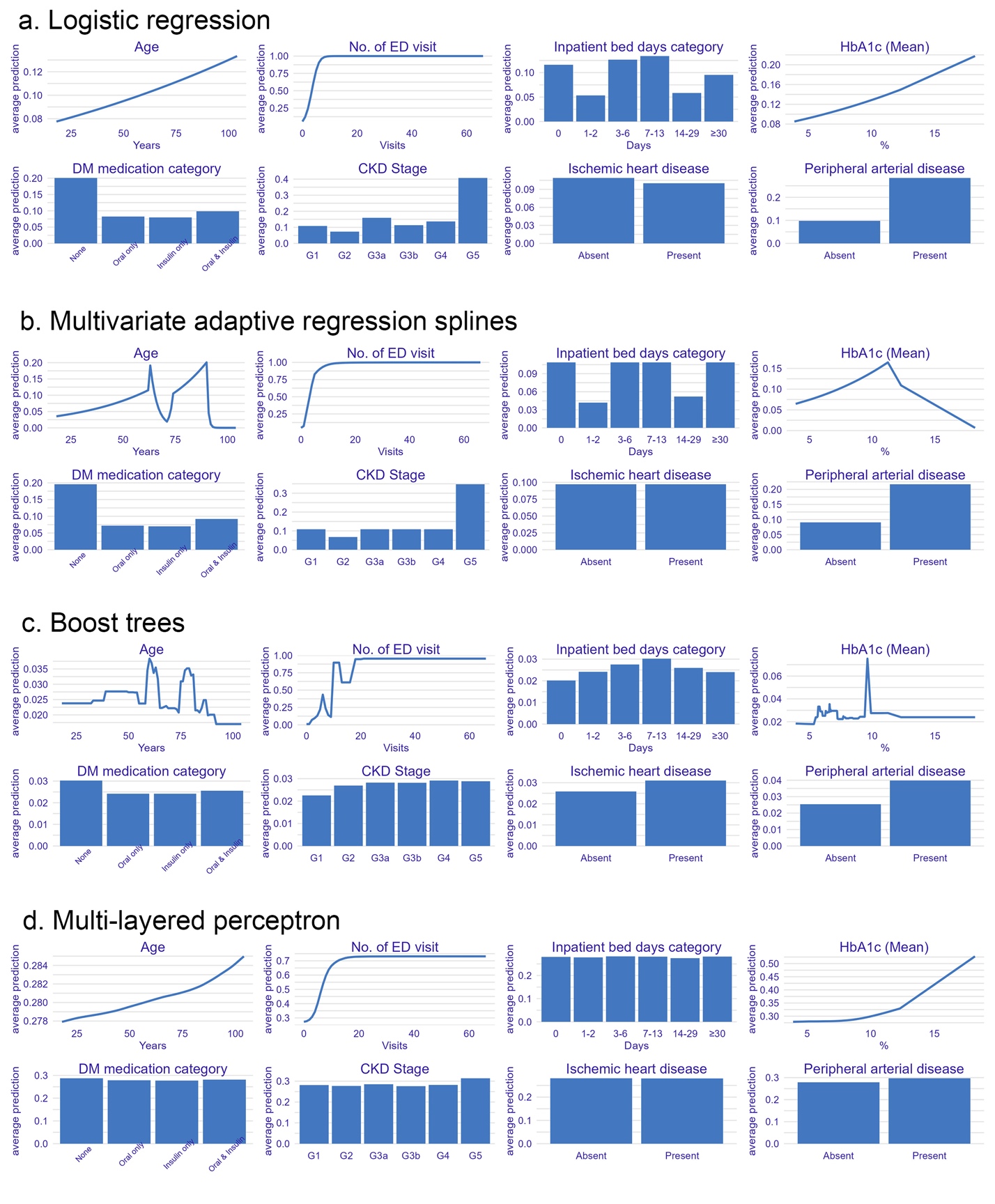

Supplement: Multimedia Appendix 1 [file ai_v3i1e58463_app1.docx]
