# Supplementary material for: Machine Learning–Based Prediction for High Health Care Utilizers by Using a Multi-Institutional Diabetes Registry: Model Training and Evaluation
Source: JMIR AI. 2024 Oct 17;3:e58463. doi: 10.2196/58463 (PMC11528163; doi:10.2196/58463)
Supplement: Multimedia Appendix 2 [file ai_v3i1e58463_app2.pdf]

Multimedia Appendix 2

Contents

Supplementary Figures
Figure 1. Comparing between different oversampling techniques to predict inpatient bed days
Figure 2. Comparison between different oversampling techniques to predict emergency department visits.
Figure 3. Performance of models trained using random oversampling to predict inpatient bed days.
Figure 4. Performance of models trained using random oversampling to predict emergency department visits.

Figure 1. Comparing between different oversampling techniques to predict inpatient bed days.

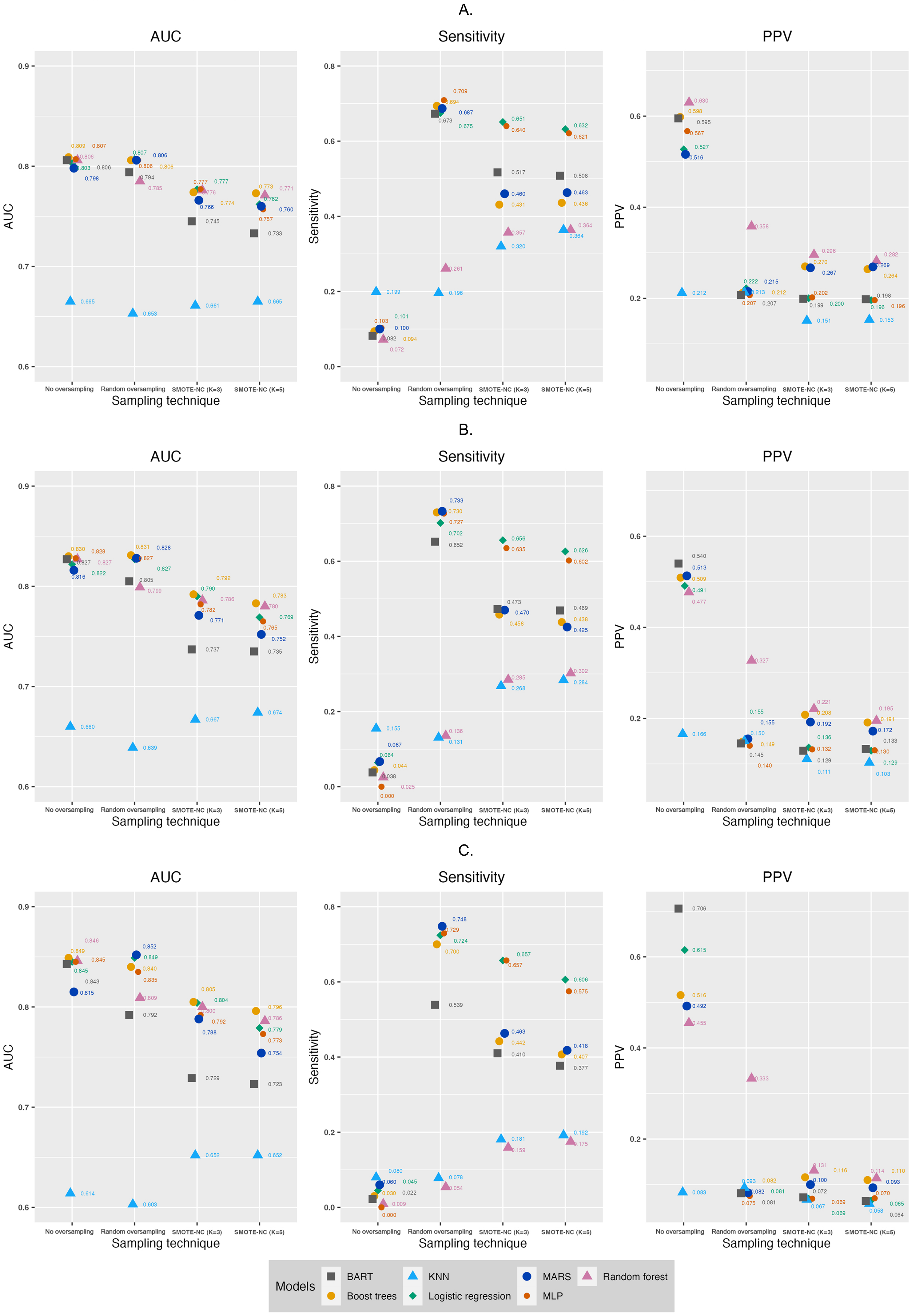

Figure 2. Comparison between different oversampling techniques to predict emergency department visits.

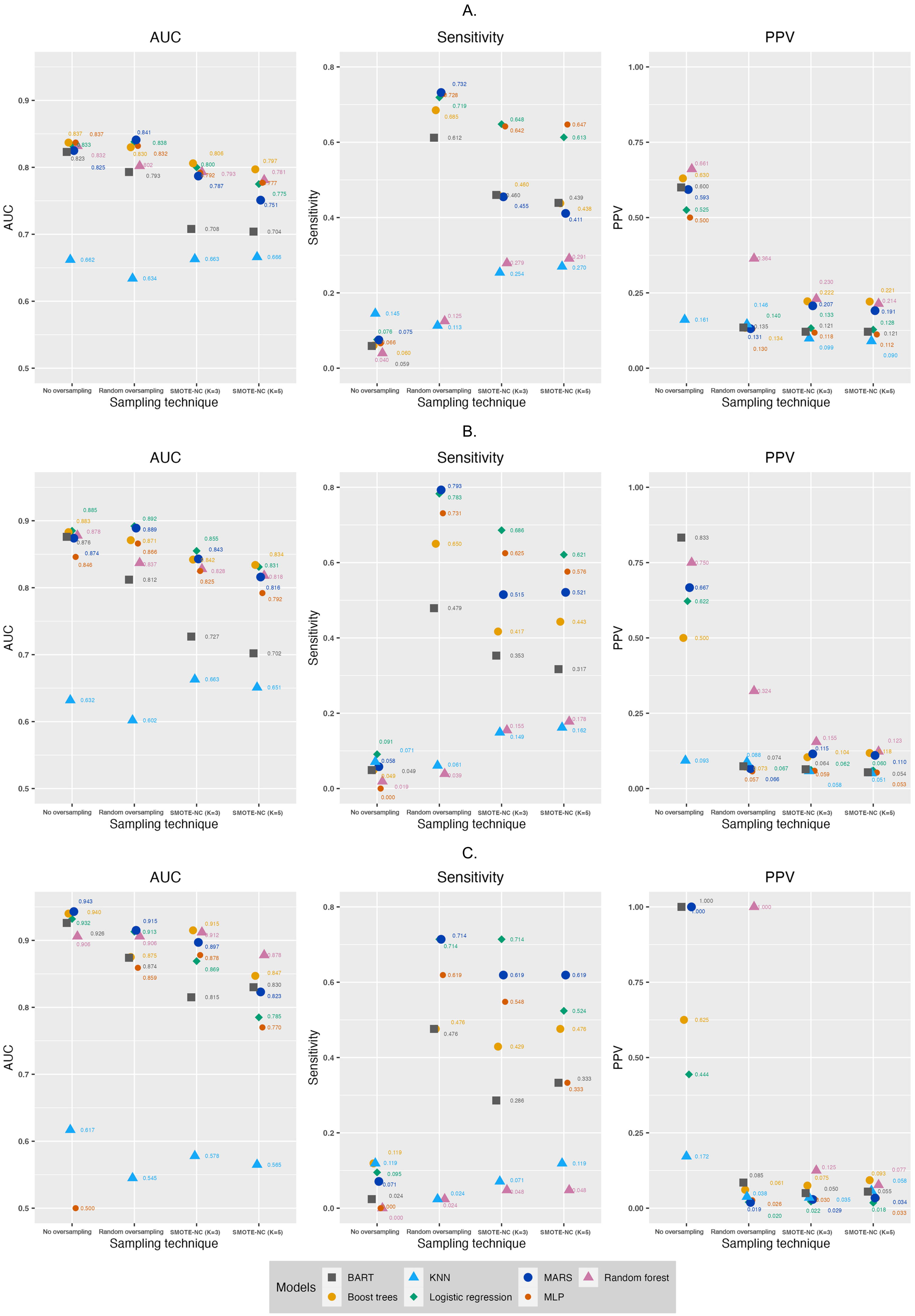

Figure 3. Performance of models trained using random oversampling to predict inpatient bed days.

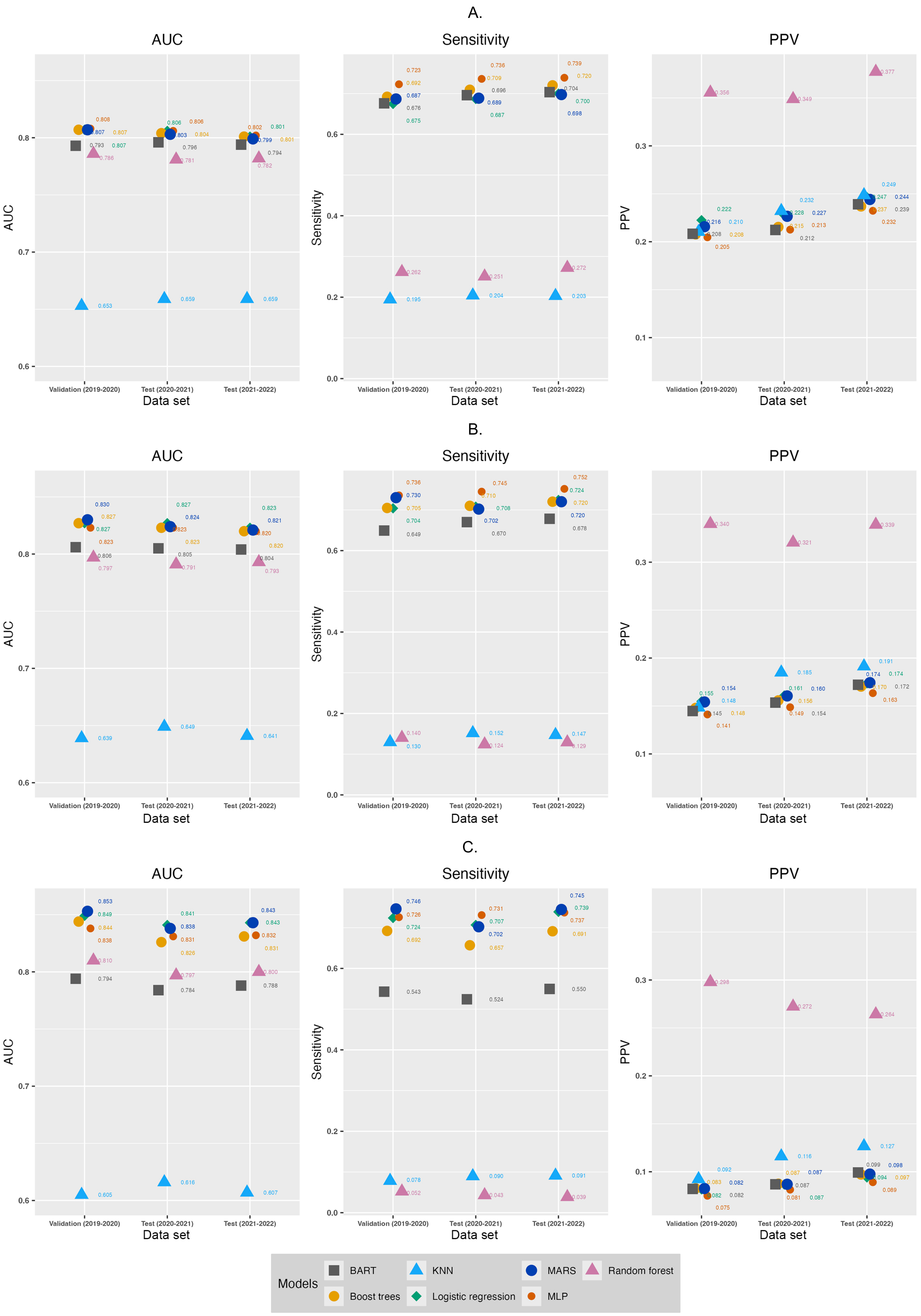

Figure 4. Performance of models trained using random oversampling to predict emergency department visits.

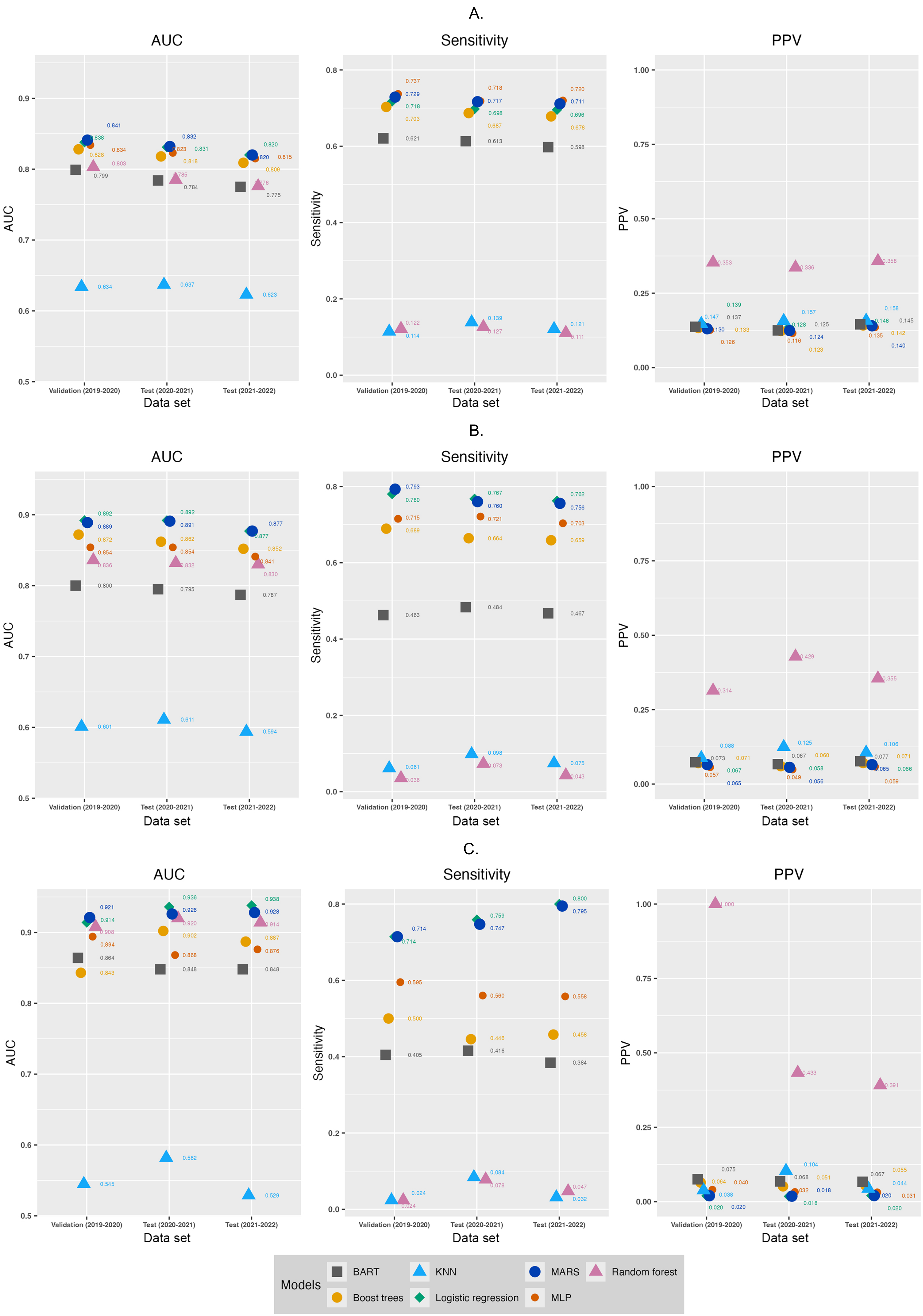

Abbreviations

AUC: area under the receiver operating curve

BART: Bayesian additive regression trees

KNN: k-nearest neighbor

MARS: multivariate adaptive regression splines

MLP: multi-layer perceptron

PPV: positive predictive value

SMOTE-NC: synthetic minority over-sampling technique-nominal continuous
